# Validity of Electronically Administered Recent Physical Activity Questionnaire (RPAQ) in Ten European Countries

**DOI:** 10.1371/journal.pone.0092829

**Published:** 2014-03-25

**Authors:** Rajna Golubic, Anne M. May, Kristin Benjaminsen Borch, Kim Overvad, Marie-Aline Charles, Maria Jose Tormo Diaz, Pilar Amiano, Domenico Palli, Elisavet Valanou, Matthaeus Vigl, Paul W. Franks, Nicholas Wareham, Ulf Ekelund, Soren Brage

**Affiliations:** 1 MRC Epidemiology Unit, University of Cambridge, Cambridge, United Kingdom; 2 Julius Center for Health Sciences and Primary Care, University Medical Center Utrecht, Utrecht, The Netherlands; 3 Department of Community Medicine, Faculty of Health Sciences, University of Tromsø, The Arctic University of Norway, Tromsø, Norway; 4 Section for Epidemiology, Department of Public Health, Aarhus University, Aarhus, Denmark and Department of Cardiology, Aalborg University Hospital, Aalborg, Denmark; 5 Inserm, Centre for research in Epidemiology and Population Health, U1018, Lifelong epidemiology of obesity, diabètes and chronic renal disease Team, F-94807, Villejuif, France; Univ Paris-Sud, UMRS 1018, F-94807, Villejuif, France; 6 Department of Epidemiology, Murcia Regional Health Authority, Murcia, Spain; 7 CIBER Epidemiología y Salud Pública (CIBERESP), Spain; 8 Department Sociosanitary Sciences, Murcia School of Medicine, Murcia, Spain; 9 Subdirección de Salud Pública de Gipuzkoa, Gobierno Vasco, San Sebastian, Spain; 10 Molecular and Nutritional Epidemiology Unit, ISPO, Cancer Prevention and Research Institute, Florence, Italy; 11 Hellenic Health Foundation (HHF), Athens, Greece; 12 Department of Epidemiology, Deutsches Institut für Ernährungsforschung Potsdam-Rehbrücke, Nuthetal, Germany; 13 Department of Clinical Sciences, Genetic & Molecular Epidemiology Unit, Skåne University Hospital, Lund University, Malmö, Sweden; 14 Genetic Epidemiology & Clinical Research Group, Department of Public Health & Clinical Medicine, Section for Medicine, Umeå University, Umeå, Sweden; Iran University of Medical Sciences, Iran (Republic of Islamic)

## Abstract

**Objective:**

To examine the validity of the Recent Physical Activity Questionnaire (RPAQ) which assesses physical activity (PA) in 4 domains (leisure, work, commuting, home) during past month.

**Methods:**

580 men and 1343 women from 10 European countries attended 2 visits at which PA energy expenditure (PAEE), time at moderate-to-vigorous PA (MVPA) and sedentary time were measured using individually-calibrated combined heart-rate and movement sensing. At the second visit, RPAQ was administered electronically. Validity was assessed using agreement analysis.

**Results:**

RPAQ significantly underestimated PAEE in women [median(IQR) 34.1 (22.1, 52.2) vs. 40.6 (32.4, 50.9) kJ/kg/day, 95%LoA: −44.4, 63.4 kJ/kg/day) and in men (43.7 (29.0, 69.0) vs. 45.5 (34.1, 57.6) kJ/kg/day, 95%LoA: −47.2, 101.3 kJ/kg/day]. Using individualised definition of 1MET, RPAQ significantly underestimated MVPA in women [median(IQR): 62.1 (29.4, 124.3) vs. 73.6 (47.8, 107.2) min/day, 95%LoA: −130.5, 305.3 min/day] and men [82.7 (38.8, 185.6) vs. 83.3 (55.1, 125.0) min/day, 95%LoA: −136.4, 400.1 min/day]. Correlations (95%CI) between subjective and objective estimates were statistically significant [PAEE: women, rho = 0.20 (0.15–0.26); men, rho = 0.37 (0.30–0.44); MVPA: women, rho = 0.18 (0.13–0.23); men, rho = 0.31 (0.24–0.39)]. When using non-individualised definition of 1MET (3.5 mlO_2_/kg/min), MVPA was substantially overestimated (∼30 min/day). Revisiting occupational intensity assumptions in questionnaire estimation algorithms with occupational group-level empirical distributions reduced median PAEE-bias in manual (25.1 kJ/kg/day vs. −9.0 kJ/kg/day, p<0.001) and heavy manual workers (64.1 vs. −4.6 kJ/kg/day, p<0.001) in an independent hold-out sample.

**Conclusion:**

Relative validity of RPAQ-derived PAEE and MVPA is comparable to previous studies but underestimation of PAEE is smaller. Electronic RPAQ may be used in large-scale epidemiological studies including surveys, providing information on all domains of PA.

## Introduction

Epidemiological studies have demonstrated that physical inactivity (PA) is an important determinant of numerous chronic diseases, including type 2 diabetes, obesity, cardiovascular disease and certain types of cancer[1]–[3]. Current evidence based on the WHO repository of the International Physical Activity Questionnaire (IPAQ) and Global Physical Activity Questionnaire (GPAQ) data suggests that approximately 30% of the population worldwide is considered insufficiently active, making physical inactivity an important public health concern [4].

PA is a complex behaviour that is difficult to assess accurately in free-living individuals [5]. Accurate and precise measurement of PA is essential for accurately estimating the effect size of PA on a particular health outcome, for making meaningful cross-cultural comparisons, for assessing the effect of interventions, and for monitoring temporal trends of PA within populations [6]. For practical reasons, physical activity questionnaires are the most commonly used assessment method in large-scale epidemiological studies [7] either as surveillance tools or in aetiological investigations. Nevertheless, questionnaires have limitations in terms of validity and reliability [8], [9] and are subject to recall and response biases [10], which must be quantified to facilitate interpretation of the information gathered. Therefore, it is important to validate any PA-questionnaire against an objective criterion measure in a population representative of that to which it will be applied.

A number of PA-questionnaires used within epidemiological studies [7] are focused on PA in only one domain, such as recreational or occupational PA, without assessing total PA. In addition, they may not capture all dimensions of PA including duration, frequency and intensity. Furthermore, the duration of sedentary time (SED-time) represents an important concept in its own right due to its associations with major chronic diseases[11]–[14]. Important attributes of a questionnaire therefore include information on both active and sedentary pursuits, in all domains. Given the complexity of retrieval of PA from the memory, it may be easier to recall specific activities rather than aggregated time spent sedentary or in moderate or vigorous PA [15] which then allows assignment of different layers of meaning to the answers given. Lastly, an implicit assumption often applied when deriving PAEE from a questionnaire is that an individual spends the entire reported time for an activity at the same intensity level, which is unlikely to be true for all activities, as intensity tends to vary between and within individuals.

The Recent Physical Activity Questionnaire (RPAQ) was designed based on the European Prospective Investigation into Cancer and Nutrition (EPIC)-Norfolk Physical Activity Questionnaire (EPAQ2) [7] and inquires about PA across four domains (leisure time, occupation, commuting, and domestic life) during the past 4 weeks [16]. An initial assessment of reliability and validity of the RPAQ was conducted on a sample of participants living in Cambridgeshire (United Kingdom) and showed moderate-to-high reliability, with an intra-class correlation coefficient (ICC) of 0.76 (p<0.001) for physical activity energy expenditure (PAEE), and good validity for ranking individuals according to their time spent in vigorous intensity PA and overall PAEE [16]. The RPAQ is currently being used in several population-based studies and interventions[17]–[28], highlighting the need to establish its validity in a larger and more heterogeneous sample.

The aims of this study were to: 1) extend the initial validation work [16] by establishing the validity of the RPAQ in larger samples of the adult population of 10 European countries using objective measurement of PA by combined accelerometry and heart rate monitoring with individual calibration as the criterion method [29]; and 2) revisit the intensity assumptions underlying the calculation of PAEE at work from self-report and to assess the impact on validity after applying these assumptions.

## Methods

### Ethics Statement

Each participating study centre obtained ethical approval from its local ethics committee.

### Study Population and Design

Full details of the study population and design have been described elsewhere [30]. Briefly, adult middle-aged men and women from 10 European countries (Denmark, France, Germany, Greece, Italy, Netherlands, Norway, Spain, Sweden, and UK) were recruited between November 2006 to February 2007. A convenience sample of approximately 200 participants was recruited in each country, representative with respect to baseline age and sex of each of the EPIC-cohorts [31]. As a consequence, only women were recruited in France and Norway. All individuals received verbal and written information about the study and provided written consent. For the purposes of this validation study two visits were held, with a median (IQR) time between the visits of 4.4 (3.9–5.0) months. Anthropometric parameters were measured at both visits according to standard clinical procedures.

### RPAQ Questionnaire

RPAQ was administered electronically at the end of the second visit, except in Sweden where a paper version was used. Standard methods of translation to all languages and back-translation to English were applied to ensure functional and conceptual equivalence of the instrument in all 10 countries. RPAQ represents a modified and shorter version of the EPAQ2 [7], with a shorter timeframe of reference (4 weeks compared with one year) and closed questions with ordered categories of bout frequency, paired with bout duration on a continuous scale. The information was collected in a disaggregated way (in contrast to IPAQ, for example), such that it may be aggregated by intensity, domain, or other constructs. The RPAQ consists of 9 main questions which cover 4 domains of PA [16]: domestic life, work, recreation and transport. The domestic PA section contains questions regarding computer use, TV-viewing and stair climbing at home. The categories of occupational PA were adopted from the Modified Tecumseh Occupational Activity Questionnaire which has been validated elsewhere [16], [32]. The questions in the recreational domain were adapted from the Minnesota Leisure Time Activity Questionnaire [33] and ask about frequently performed activities [34]. Commuting includes 4 modes of usual transport: walking, cycling, car, and public transportation. The English version of RPAQ including the syntax for interpretation is available from www.mrc-epid.cam.ac.uk/research/resources [35].

Summary variables from the RPAQ were derived according to the methods described previously [16]. However, there were slight differences in the version of RPAQ used in this validation study. Information on the number of working hours for each of the four weeks and distance from home to work was directly asked in the current version questionnaire, so as to avoid assumptions regarding those parameters, and countries were allowed to add additional leisure activities that were common in each location. Estimates of PAEE for each activity were calculated for all 4 domains (leisure, work, commuting and domestic life) by multiplying the duration of each activity (h/day) with its metabolic cost in metabolic equivalent tasks (MET) which was obtained from the Compendium of Physical Activities [36] using the calculations that have been described in detail earlier [16].

All activities were categorised with respect to intensity as follows: sedentary (<1.5 MET); light (1.5 to <3 MET), moderate (3 to 6 MET) and vigorous (>6 MET), whereby the latter two categories were combined in one category which is referred to as moderate-to-vigorous and includes activities >3MET. Occupations were classified into 4 groups, which were scored according to presumed average intensity: sitting (1.5 MET); standing (2.3 MET); manual (3.5 MET); and heavy manual (5.5 MET). Self-reported sedentary time was calculated as the sum of time spent watching TV, using computer, using motorised transportation, sleep, and sitting time at work among those who reported a sedentary type of job.

To compare our results with the validity of the Short EPIC-PA-questionnaire [30], which was examined in the same sample, we derived the Cambridge Index based on the information on occupational category (sitting, standing, manual and heavy manual) and time spent in sports and cycling. The Cambridge Index classifies individuals in four categories: inactive, moderately inactive, moderately active and active [30].

### Objective Physical Activity Measurement Methods

PA was objectively measured using a combined heart rate (HR) and acceleration monitor (Actiheart, CamNtech Ltd, Cambridge, UK) attached to the participants’ chest by two standard electrocardiogram electrodes, as described previously [29]. All participants performed an eight-minute ramped step test using a 200 mm step (Reebok, Lancaster, UK) to assess the individual relationship between HR and work load [37].

Having completed the step test, the combined HR and movement sensor was re-initialised to collect data minute-by-minute and participants were asked to wear the sensor for 24 h/day for a minimum of 4 days. HR data were processed using Gaussian process robust regression to deal with measurement noise [38] and accelerometry data were analysed in its raw form. Activity intensity (J/min/kg) was estimated from the combination of movement registration and individually calibrated HR [37] using a branched equation model [39]. Periods of inactivity lasting >60 min accompanied by non-physiological HR were treated as non-wear and were taken into account to minimise diurnal information bias when summarising intensity time-series into PAEE (kJ/kg/day) and time spent in sedentary (SED-time, h/day) and moderate-to-vigorous intensity PA (MVPA, min/day). Individual records with less than 24 h of wear data were excluded. Furthermore, PAEE, MVPA and SED-time were weighted according to the duration of monitoring when averaged from the 2 visits. SED-time was considered as time spent at intensity of ≤1.5 MET [40].

We generated two sets of intensity variables from the objective monitoring records: one based on an individualised definition of 1 MET as estimated by the Oxford equations [41] for resting metabolic rate (RMR), and the other based on the standard definition [36] of 1 MET = 3.5 mlO_2_/min/kg (Supplementary material). The same factorial cut-offs were used in both sets of variables, with intensity ≤1.5 MET representing sedentary behaviour, 1.5 to <3.0 MET light PA, and ≥3.0 MET indicating MVPA.

To revisit the assumptions underlying the calculation of PAEE in occupational domain from the questionnaire, we applied empirically-derived PA-intensity distribution to the questionnaire data, and recalculated PAEE. To achieve this, we first randomly split the sample into two sub-samples containing 2/3 (“training sample”) and 1/3 (validation “holdout sample”) of all participants in each of the four occupational categories. To determine the empirical intensity distribution of work and allowing for cultural differences in working pattern, we selected person-hours with valid monitor data between 10∶00 and 15∶00 on weekdays (Monday-Friday). We summarised the proportion of time spent at 18 narrowly defined intensity categories (1.25 to 11+ METs, with higher resolution at the lower end of spectrum) in the “training sample”, and applied it to the self-reported work duration in the “holdout sample” to recalculate PAEE and assess impact on validity.

### Statistical Analysis

Participant characteristics are presented as means and standard deviations for continuous variables and frequencies with percentages for categorical variables.

Absolute validity of the RPAQ-estimate of PAEE, MVPA and SED-time was assessed by the degree of agreement with criterion measures according to the Bland-Altman technique [42]. Due to skewness, however, we present median (IQR) for PA-variables, and median biases, defined as the median difference between objective and self-reported estimates, with 95% limits of agreement (LoA) presented as the 2.5^th^ and 97.5^th^ percentile of the difference. Spearman’s correlation coefficients (rho) were used to examine heteroscedasticity from Bland-Altman plots. To examine the differences in bias by BMI-category and employment status Kruskal-Walis and Mann-Whitney tests were used, respectively. To examine whether validity differed by age, sex, BMI and employment status, we included interaction terms between these variables and self-reported PA on objectively assessed PA. The corresponding interaction terms were added in separate linear regression models and significance of interaction term was tested. Although interactions with sex were not statistically significant, we decided a priori to present the results stratified by sex for comparability with the studies that included only men or women.

The associations between objective and subjective estimates of PAEE, MVPA and SED-time were assessed by Spearman’s correlation coefficients (rho) for each country. These were Fisher transformed and analysed in random-effects meta-analysis to calculate the combined correlation across the countries. Heterogeneity in the association between questionnaire-derived and objective estimates across the countries was assessed by the *I^2^*-statistic. Partial correlation coefficients were calculated to assess the correlation of domain-specific PAEE derived from the questionnaire with objectively measured PAEE adjusted for the other 3 PA-domains. Spearman’s correlation coefficients were calculated to assess the relationship of RPAQ-derived PAEE and MVPA with the 4-category Cambridge Index [30]. Multivariate test for means was conducted to examine the differences between objectively measured intensity distributions across the four occupational groups. The analyses were performed using STATA version 12 (STATA Corp, College Station, Texas). All statistical tests were two-sided, with a threshold for statistical significance set at p<0.05.

## Results

Participant baseline characteristics stratified by country and sex are shown in Table 1. Of the 1,923 participants, 69.8% were women and 76.2% were employed. Median (IQR) duration of monitor wear was 4.4 (4.0–5.9) days during the first measurement period and 4.5 (4.0–5.9) days during the second measurement period. No significant interactions were found for the 3 PA-subcomponents of interest (PAEE, MVPA and SED-time) with age, sex, BMI and employment status, with exception of a statistically significant interaction with BMI when objectively measured SED-time was derived using standard definition of 1MET (p = 0.005).

**Table 1:** Participant characteristics at baseline, the RPAQ validation study cohort (N = 1923).

Country	Sex	N	Age (years)	Weight (kg)	Height (m)	BMI (kg/m^2^)	WHR	Employed
Denmark	Women	113	57.6 (4.2)	69.8 (12.9)	1.64 (0.05)	25.7 (4.5)	0.82 (0.07)	86 (76.1)
	Men	67	58.4 (3.6)	87.2 (10.6)	1.78 (0.06)	27.7 (3.3)	0.98 (0.18)	53 (79.1)
France	Women	174	54.7 (7.5)	61.3 (9.4)	1.62 (0.06)	23.2 (3.3)	0.77 (0.06)	143 (82.2)
Germany	Women	125	55.4 (4.5)	69.1 (11.1)	1.64 (0.06)	25.6 (4.1)	0.82 (0.06)	100 (80.0)
	Men	83	58.0 (3.1)	86.2 (12.5)	1.77 (0.05)	27.6 (3.5)	0.96 (0.06)	66 (79.5)
Greece	Women	121	51.7 (16.0)	69.2 (13.1)	1.61 (0.06)	27.0 (5.4)	0.84 (0.07)	64 (52.9)
	Men	67	50.5 (18.8)	84.8 (12.5)	1.75 (0.08)	27.8 (3.7)	0.95 (0.07)	44 (65.6)
Italy	Women	142	52.9 (6.5)	63.4 (10.4)	1.60 (0.06)	24.9 (3.8)	0.78 (0.07)	111 (78.2)
	Men	53	53.3 (6.6)	78.9 (13.6)	1.73 (0.06)	26.9 (4.0)	0.91 (0.07)	42 (79.3)
Netherlands	Women	179	58.9 (10.4)	62.7 (7.9)	1.67 (0.07)	22.6 (2.4)	0.83 (0.06)	111 (62.0)
	Men	30	50.5 (11.2)	77.9 (9.9)	1.82 (0.06)	23.5 (2.2)	0.87 (0.05)	24 (80.0)
Norway	Women	177	48.2 (6.7)	70.8 (10.7)	1.65 (0.06)	26.0 (3.5)	0.84 (0.06)	154 (87.0)
Spain	Women	113	49.1 (8.4)	65.1 (9.9)	1.60 (0.06)	25.5 (3.7)	0.81 (0.07)	82 (71.9)
	Men	92	51.5 (7.2)	80.4 (11.0)	1.72 (0.07)	27.1 (3.4)	0.94 (0.06)	80 (87.0)
Sweden	Women	95	52.4 (8.5)	72.1 (12.8)	1.65 (0.06)	26.6 (4.9)	0.87 (0.06)	81 (87.1)
	Men	92	52.4 (7.9)	84.9 (13.3)	1.79 (0.07)	26.5 (3.6)	0.96 (0.07)	82 (90.1)
UK	Women	104	59.8 (7.6)	70.0 (11.4)	1.62 (0.06)	26.7 (4.1)	0.86 (0.06)	60 (57.7)
	Men	96	61.4 (7.9)	85.6 (12.9)	1.76 (0.06)	27.7 (3.4)	0.96 (0.07)	59 (61.5)
Total	Women	1343	54.0 (9.3)	66.9 (11.5)	1.63 (0.07)	25.2 (4.2)	0.82 (0.07)	992 (74.0)
	Men	580	55.0 (9.9)	83.9 (12.4)	1.76 (0.07)	27.0 (3.6)	0.95 (0.09)	450 (77.7)

Values are mean (SD), except employment status which is n (%).

Abbreviations: BMI- body mass index; WHR- waist-hip ratio;

p<0.001 for difference in all variables across the countries (Kruskal-Wallis test).

**Figure 1:**
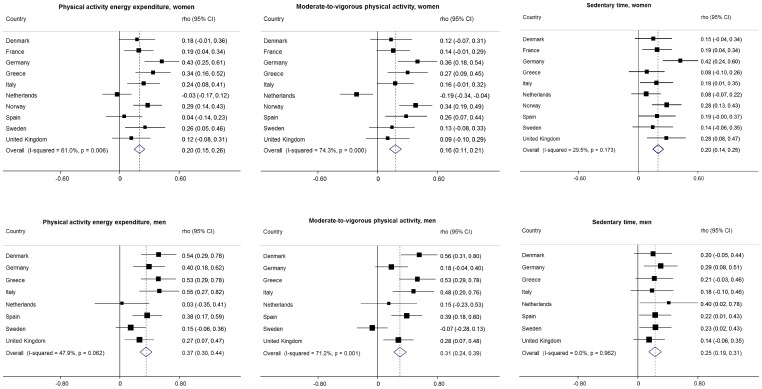
Spearman’s correlation coefficients for the associations of PAEE, MVPA and sedentary time assessed by the RPAQ with objectively measured corresponding variables by country and sex in 1343 women and 540 men.

Tables 2–4 show questionnaire-derived and objective estimates of PAEE, MVPA and SED-time, respectively, using individualised definition of 1MET. The corresponding values from the analysis with standard definition of 1MET are given in Supplementary tables 1 and 2 (not applicable to PAEE). Figure 1 shows Spearman’s correlation coefficients comparing questionnaire-derived with criterion-measured PAEE, MVPA and SED-time by country and sex.

**Table 2:** Physical activity energy expenditure (kJ/kg/day) as assessed by the Recent Physical Activity Questionnaire and combined movement sensor and heart rate monitor, N = 1923.

	RPAQ					Acc+HR					Inter-method difference			
	Mean	SD	Median	IQR		Mean	SD	Median	IQR		Mean bias	Median bias	LoA	
Women, N = 1343														
Denmark	53.8	35.6	47.4	27.0	68.4	39.0	11.8	38.0	30.2	47.2	14.8	6.0***	−28.9	104.1
France	34.2	20.9	29.7	19.6	43.8	38.2	11.9	37.5	30.8	45.4	−4.0	−8.9*	−33.8	50.8
Germany	46.2	35.2	36.7	24.5	54.8	40.7	14.3	38.7	30.0	49.7	5.5	−1.0	−30.0	64.0
Greece	28.6	21.6	21.9	13.6	39.2	38.8	14.1	38.2	29.7	48.1	−10.2	−14.0***	−43.0	36.5
Italy	31.5	18.8	25.7	18.8	39.2	46.3	13.6	44.3	36.8	55.5	−14.8	−16.6	−50.6	23.4
Netherlands	57.2	26.1	51.5	40.4	72.4	46.5	17.6	43.6	35.4	54.9	10.7	8.6***	−44.9	81.2
Norway	40.8	25.2	35.1	23.1	53.0	45.1	14.5	42.7	35.1	53.7	−4.3	−7.4*	−48.7	58.7
Spain	32.9	17.3	29.6	21.1	42.6	47.9	13.5	46.0	38.6	57.1	−15.0	−15.3***	−51.0	30.9
Sweden	40.8	30.6	33.7	21.9	49.6	42.9	14.6	41.0	32.1	51.7	−2.1	−7.5	−36.0	79.0
United Kingdom	36.3	22.3	29.2	19.1	49.9	35.3	11.6	33.5	26.4	44.1	1.0	−3.3	−36.8	54.6
Total, women	40.6	27.3	34.1	22.1	52.2	42.4	14.5	40.6	32.4	50.9	−1.7	−6.5*	−44.4	63.4
Men, N = 580														
Denmark	71.2	61.0	52.8	38.8	80.6	43.1	17.4	41.5	32.4	51.8	28.1	13.1***	−26.5	232.9
Germany	62.3	39.4	51.0	35.8	78.1	41.6	13.9	41.0	31.3	49.4	20.7	14.0***	−27.9	94.6
Greece	40.5	30.0	30.6	22.4	45.7	44.2	19.3	43.8	29.4	54.7	−4.7	−7.3	−45.7	63.9
Italy	44.5	25.9	42.0	28.2	54.4	51.2	15.8	48.7	41.5	60.1	−6.7	−12.1*	−37.4	44.7
Netherlands	56.8	24.4	54.2	38.5	66.3	55.2	15.1	54.0	44.6	63.7	1.6	0.7	−63.7	74.3
Spain	50.2	34.8	44.7	28.5	59.4	51.1	19.5	48.6	38.2	62.5	−0.5	−5.1	−47.8	55.1
Sweden	52.1	34.5	43.3	27.7	68.9	55.3	18.8	52.4	41.3	67.0	−2.9	−8.6	−64.2	101.3
United Kingdom	61.0	72.7	47.5	25.9	74.3	40.0	16.2	36.6	29.3	48.9	21.0	7.9*	−39.4	105.5
Total, men	55.2	46.5	43.7	29.0	69.0	47.1	18.2	45.5	34.1	57.6	8.1	1.1***	−47.2	101.3
Total, both sexes	34.8	55.2	37.7	23.6	45.0	43.6	15.8	46.5	32.7	53.3	1.2	−4.6	−46.0	78.7

IQR- interquartile range; SD- standard deviation; LoA- 95% limits of agreement; range of bias includes the values between 2.5^th^ and 97.5^th^ percentile;

Acc+HR- combined accelerometer and heart rate monitor.

*p<0.05, **p<0.01, ***p<0.001 for bias.

**Table 3:** Time spent in moderate and vigorous physical activity (min/day) as assessed by the Recent Physical Activity Questionnaire and combined movement sensor and heart rate monitor, N = 1923.

	RPAQ					Acc+HR					Inter-method difference			
	Mean	SD	Median	IQR		Mean	SD	Median	IQR		Mean bias	Median bias	LoA	
Women, N = 1343														
Denmark	107.4	100.0	71.8	46.1	120.0	72.5	37.4	63.5	45.2	95.5	34.8	3.1***	−90.3	322.0
France	71.9	84.1	48.6	23.4	86.8	71.6	37.6	65.4	46.3	88.8	0.3	−17.1	−122.0	262.9
Germany	106.5	97.4	74.0	41.9	133.9	80.1	46.1	73.0	46.1	100.3	26.5	7.1**	−117.0	294.2
Greece	69.2	92.5	41.4	19.8	72.6	70.5	40.6	68.1	42.9	92.3	−1.4	−15.1	−102.0	275.0
Italy	78.2	91.5	41.9	18.5	103.9	80.4	52.3	72.5	46.0	101.0	−2.2	−21.5	−137.9	264.4
Netherlands	169.1	105.1	146.3	99.1	205.7	97.6	57.8	90.0	57.3	124.0	71.5	61.2***	−101.2	368.2
Norway	100.8	118.3	53.6	24.4	114.8	91.0	53.8	79.2	54.8	119.2	9.8	−22.5	−130.5	348.8
Spain	70.4	59.3	56.4	26.2	99.9	96.4	49.5	89.2	58.4	125.6	−26.0	−30.4**	−143.3	163.3
Sweden	73.0	97.7	38.6	22.9	68.2	91.2	44.9	85.1	55.1	120.8	−18.2	−39.7	−163.8	305.3
United Kingdom	86.8	88.0	58.0	35.2	101.7	60.5	40.1	50.9	29.2	84.1	26.3	5.5**	−110.3	271.7
Total, women	96.4	100.6	62.1	29.4	124.3	81.9	48.6	73.6	47.8	107.2	14.4	−8.5***	130.5	305.3
Men, N = 580														
Denmark	155.8	144.5	100.7	45.0	210.1	82.3	53.3	75.0	42.3	112.4	73.5	26.9***	−108.7	399.9
Germany	175.5	166.8	112.5	49.8	257.6	83.8	43.1	77.5	50.5	109.8	91.7	41.1***	−98.1	427.3
Greece	84.4	121.0	39.7	10.7	98.0	96.4	89.1	82.5	38.3	137.2	−12.0	−26.5	−132.1	285.4
Italy	101.3	113.3	53.4	38.0	120.3	86.1	42.2	81.0	56.4	113.5	15.1	−13.6	−124.1	385.7
Netherlands	116.1	88.2	95.9	52.2	148.1	110.2	47.3	101.8	80.9	138.5	5.9	−4.6	−195.3	283.3
Spain	120.1	152.4	74.3	35.6	140.0	101.3	59.6	92.7	62.8	139.0	18.8	−8.0	−146.1	334.3
Sweden	153.2	158.9	68.8	36.1	292.8	128.2	70.2	111.6	73.7	166.3	24.9	−24.8	−224.9	429.7
United Kingdom	184.5	168.1	122.2	49.8	293.2	79.4	62.2	64.1	41.5	115.3	105.1	51.7***	−83.5	534.5
Total, men	142.0	151.5	82.7	38.8	185.6	95.9	63.4	83.3	55.1	125.0	46.1	5.5	−136.4	400.1
Total, both sexes	103.3	119.5	60.7	26.2	130.4	85.4	53.4	75.7	48.2	11.4	18.4	−4.7***	−137.8	348.8

IQR- interquartile range; SD- standard deviation; LoA- 95% limits of agreement; range of bias includes the values between 2.5^th^ and 97.5^th^ percentile;

Acc+HR- combined accelerometer and heart rate monitor.

*p<0.05, **p<0.01, ***p<0.001 for bias.

**Table 4:** Time spent sedentary (h/day) as assessed by the Recent Physical Activity Questionnaire and combined movement sensor and heart rate monitor, N = 1923.

	RPAQ					Acc+HR					Inter-method difference			
	Mean	SD	Median	IQR		Mean	SD	Median	IQR		Mean bias	Median bias	LoA	
Women, N = 1343														
Denmark	12.8	2.7	12.1	10.5	14.6	16.3	2.0	15.7	14.5	17.4	−3.5	−3.5***	−9.7	3.4
France	13.5	2.9	13.3	11.0	15.4	16.9	1.9	16.8	15.6	18.3	−3.5	−3.7***	−8.7	5.9
Germany	13.6	2.8	13.2	11.1	15.9	16.2	2.3	16.9	15.0	18.2	−2.6	−3.0***	−9.0	3.6
Greece	12.8	3.4	11.9	10.5	14.3	16.1	2.5	16.5	14.6	18.3	−3.3	−4.4***	−10.3	5.4
Italy	12.7	2.6	12.9	10.3	14.9	14.8	2.2	14.8	13.3	16.4	−2.1	−2.3***	−8.6	3.9
Netherlands	12.2	2.2	11.8	10.6	13.6	15.8	2.0	15.7	14.3	17.0	−3.6	−3.6***	−8.6	3.0
Norway	13.1	2.6	12.5	10.9	15.2	15.6	2.0	15.8	14.3	17.2	−2.6	−2.8***	−8.6	3.3
Spain	12.9	2.8	12.6	10.5	15.0	14.8	2.0	15.3	12.8	16.3	−1.9	−2.2***	−9.0	6.1
Sweden	12.4	2.8	11.5	10.4	14.0	15.8	2.1	15.5	14.1	17.3	−3.4	−3.2***	−9.4	3.6
United Kingdom	12.9	2.4	12.6	11.0	14.0	16.5	2.0	16.6	15.1	17.9	−3.7	−4.1***	−9.0	2.5
Total, women	12.9	2.7	12.4	10.7	14.8	15.9	2.2	15.9	14.3	17.5	−3.0	−3.3***	−9.0	4.1
Men, N = 580														
Denmark	13.2	2.9	12.1	10.8	15.7	16.2	2.4	16.1	14.2	17.4	−3.0	−3.0***	−8.8	4.9
Germany	13.9	3.0	12.9	11.7	16.2	16.4	2.2	15.9	14.8	17.4	−2.5	−2.6***	−8.1	6.0
Greece	14.1	3.9	13.0	11.0	17.5	16.1	2.8	15.7	14.2	17.8	−2.0	−2.2**	−9.5	7.5
Italy	13.2	2.9	13.2	10.7	15.1	14.7	2.3	15.0	13.6	16.1	−1.6	−1.9***	−7.4	4.5
Netherlands	14.5	2.9	13.5	12.1	17.3	15.7	2.0	15.4	14.6	17.2	−1.3	−1.2*	−7.6	3.3
Spain	13.6	2.8	13.9	11.3	16.0	15.2	2.6	15.2	13.8	16.7	−1.6	−1.4***	−7.9	4.7
Sweden	12.5	3.0	11.6	10.1	14.5	14.5	2.5	14.7	13.2	16.2	−1.9	−2.3***	−7.8	5.8
United Kingdom	13.2	2.8	12.4	11.2	14.4	16.4	2.4	16.2	14.7	18.3	−3.2	−2.9***	−9.1	5.9
Total, men	13.4	3.1	12.8	11.0	15.9	15.6	2.6	15.5	14.1	17.2	−2.2	−2.3***	−8.3	5.5
Total, both sexes	12.7	3.0	12.2	10.5	14.8	15.8	2.3	15.8	14.3	17.4	−3.1	−3.3***	−9.6	4.9

IQR- interquartile range; SD- standard deviation; LoA- 95% limits of agreement; range of bias includes the values between 2.5^th^ and 97.5^th^ percentile;

Acc+HR- combined accelerometer and heart rate monitor.

*p<0.05, **p<0.01, ***p<0.001 for bias.

### Physical Activity Energy Expenditure

#### Absolute validity

The RPAQ underestimated PAEE in women, with a significant median bias (LoA) of −6.5 (−44.4, 63.4) kJ/kg/day, corresponding to −16% of median PAEE (Table 2). In men, median bias (LoA) was positive at 1.1 (−47.2, 101.3) kJ/kg/day (about 2% of objective median), despite median self-reported PAEE being slightly lower than objective median PAEE. Median bias (LoA) for all participants was −4.6 (−46.0, 78.7) kJ/kg/day (−11.0%), which was not significantly different from 0. Notably higher RPAQ-derived PAEE in the Netherlands compared with other countries is a result of higher leisure-time-PA due to greater proportion of participants reporting high, though theoretically possible, frequencies and durations of certain activities (e.g. 9 h of do-it-yourself every day or 4 h of competitive cycling 5 times per week).

Furthermore, we examined the variation of bias by BMI-category and employment status. We found a significant difference in bias across BMI-categories (p<0.001), with an underestimation of PAEE in normal weight and overweight individuals and overestimation in the obese (data not shown). There was a substantially greater underestimation of PAEE and SED-time in the unemployed compared with the employed participants (p<0.001).

Bland-Altman plots suggest appreciable individual differences in the assessment of PAEE (Supplementary figures 1 and 2). Additionally, magnitude of error increased with increasing inter-method mean PAEE (Spearman’s correlation coefficients rho = 0.41, and rho = 0.42 in women and men, respectively, both p<0.001). However, an opposite direction of this association was noted when difference was plotted against the criterion (not shown).

**Figure 2:**
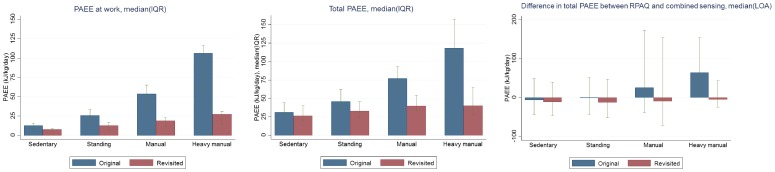
Total PAEE and PAEE at work derived from the RPAQ, and bias for total PAEE before and after applying intensity distribution assumption. Results are based on the“holdout sample”, with the following number of participants in each category: sedentary N = 264, standing N = 147, manual N = 58, heavy manual N = 12, representing 1/3 of participants in each group of the employed participants. Data are median (IQR), and median with 95% limits of agreement for bias.

#### Relative validity

A significant but weak inter-method correlation was observed for PAEE (Figure 1) in women, with a pooled estimate rho = 0.20 (95% CI: 0.15 to 0.26), and significant heterogeneity (*I^2^* = 61.0%, p = 0.006). The pooled estimate in men was greater than that in women (p = 0.003), rho = 0.37 (95% CI: 0.30 to 0.44) with borderline significant heterogeneity by country (*I^2^* = 47.9%, p = 0.062).

### Time in Moderate-to-vigorous Physical Activity

#### Absolute validity

When using individualised RMR to define objective MVPA, the RPAQ significantly underestimated MVPA (Table 3) in women with median bias (LoA) −8.5 (−130.5, 305.3) min/day (−11.5%), and significantly overestimated in men, with median bias (LoA) 5.5 (−136.4, 400.1) min/day (6.6%). There was a material underestimation in both sexes combined, with median bias (LoA) −4.7 (−137.8, 348.8) min/day (−6.2%). The observed overestimation of MVPA in men despite lower median MVPA from RPAQ than from combined sensing is a consequence of a positively skewed distribution. However, the direction of bias in MVPA varied by country in both sexes (Table 3).

Bias for MVPA did not vary by BMI-category for individualised MET estimates but when standard definition of 1MET was used in the derivation of objective intensity variables, the inter-method difference in MVPA substantially increased with BMI (p = 0.008), with the greatest overestimation among the obese. No difference in bias in MVPA was found between employed and unemployed individuals.

There were substantial individual differences in the assessment of MVPA as displayed in Bland-Altman plots (Supplementary figures 1 and 2), with an indication of proportional error (Spearman’s correlation coefficients between the difference and the average were rho = 0.36 and rho = 0.51 in women and men, respectively, and both p<0.001). Nevertheless, individuals with lower objectively measured MVPA tended to over-report MVPA to a greater extent than their more active counterparts (not shown). We found an overestimation of MVPA by RPAQ in women and men (Supplementary table 1) when standard definition of 1MET was applied.

#### Relative validity

Inter-method correlation for MVPA (Figure 1) was slightly weaker than that observed for total PAEE and greater for men than women, p = 0.003 (rho = 0.18, 95% CI: 0.13 to 0.23; *I^2^* = 64.0%, p = 0.003 for women and rho = 0.31, 95% CI: 0.24 to 0.39; *I^2^* = 71.2%, p = 0.001 for men). Comparative pooled correlation coefficients using the standard definition of 1MET were rho = 0.16, 95% CI: 0.11 to 0.21; *I^2^* = 74.3%, p<0.001 in women, and rho = 0.27, 95% CI: 0.19 to 0.34; *I^2^* = 74.1%, p<0.001 in men (Supplementary figure 3); p = 0.007 for the difference in rho between the sexes.

### Sedentary Time

#### Absolute validity

The RPAQ significantly underestimated SED-time, with median bias (LoA) −3.3 (−9.0, 4.1) h/day among women (−20.8%), and −2.3 (−8.3, 5.5) h/day (−14.8%) among men (Table 4).

Bias for SED-time did not differ across BMI-categories and employment status, but when standard definition of 1MET was used, underestimation of SED-time increased with BMI-category (p = 0.018), and was the greatest in the obese (−26%).

Assessment of SED-time varied considerably between participants (Supplementary figures 1 and 2). Magnitude of error tended to increase with greater SED-time (Spearman’s correlation coefficients rho = 0.21 and rho = 0.18 in women and men, respectively, both p<0.001). However, the tendency to under-report was associated with greater objectively measured SED-time (not shown). Bias for SED-time remained similar after using standard definition of 1MET (Supplementary table 2).

#### Relative validity

The correlation between self-reported and objectively measured SED-time (Figure 2) was comparable to that of PAEE and MVPA without substantial heterogeneity in women (rho = 0.20 (95% CI: 0.14 to 0.25), *I^2^* = 29.5%, p = 0.173). The corresponding pooled estimate in men was a rho = 0.25 (95% CI: 0.19 to 0.31), and there was no evidence of heterogeneity between the countries, *I^2^* = 0%, p = 0.962. When using the standard definition of 1MET, pooled estimate was rho = 0.19 (95% CI: 0.14 to 0.24), *I^2^* = 42.8%, p = 0.072 in women and rho = 0.22 (95% CI: 0.13 to 0.30), *I^2^* = 0%, p = 0.949 in men.

### Domain-specific PAEE from the RPAQ and Total Objectively Assessed PAEE

Domain-specific PAEE from the RPAQ (Table 5) materially differed by country in all 4 domains in both sexes. The highest PAEE was reported in the occupational domain, with median (IQR) 14.7 (10.2, 15.1) kJ/kg/day in women, and 18.3 (12.3, 36.5) kJ/kg/day in men. Partial correlations between domain-specific PAEE from the RPAQ and total objectively measured PAEE are shown in Table 5. After adjustment for all other domains, correlation coefficients varied by country, and overall there was a weak positive correlation for the occupational domain (women: r = 0.16; men: r = 0.30), leisure-time-PA (women: r = 0.13; men: r = 0.19) and commuting PA (women: r = 0.11; men: r = 0.10) but a weak negative correlation for PAEE in the home domain (women; r = −0.13; men: r = −0.11). However, none of these partial correlations was statistically significant, although there was evidence of significance in some countries in every PA-domain.

**Table 5:** Domain-specific energy expenditure from the RPAQ and partial correlation with objectively assessed physical activity energy expenditure adjusted for all other domains (580 men and 1343 women).

	PAEE for leisure(kJ/kg/day)					PAEE at work(kJ/kg/day)					PAEE for commuting(kJ/kg/day)					PAEE at home(kJ/kg/day)				
	Median	IQR		r	p-valuefor r	Median	IQR		r	p-valuefor r	Median	IQR		r	p-valuefor r	Median	IQR		r	p-valuefor r
Women, N = 1343																				
Denmark	15.3	10.8	24.5	0.12	0.010	15.9	12.5	32.0	0.06	<0.001	0.9	0	2.8	0.12	0.375	4.1	2.5	5.8	−0.25	0.005
France	9.3	5.4	18.3	0.11	0.138	13.9	9.2	20.4	0.21	0.006	0.4	0	1.3	0.10	0.175	3.5	2.1	5.7	−0.24	0.002
Germany	14.8	8.2	29.4	0.22	0.004	16.3	11.5	23.2	0.19	0.002	0.6	0.1	1.7	0.11	0.008	3.2	1.9	5.0	−0.23	0.085
Greece	8.5	3.6	14.6	0.18	<0.001	15.0	10.1	29.2	0.31	<0.001	0.0	0	0.4	0.01	0.210	3.0	1.7	4.7	−0.13	0.508
Italy	7.9	3.5	19.0	0.23	<0.001	13.7	10.9	20.7	0.31	<0.001	0.4	0	1.7	−0.01	0.960	2.0	1.2	3.6	0.28	0.043
Netherlands	37.9	26.4	58.1	−0.03	0.549	8.2	3.6	14.6	0.08	0.254	0.5	0	4.3	0.13	0.031	3.8	2.4	5.7	−0.17	0.043
Norway	9.6	4.4	16.8	0.30	<0.001	15.9	11.6	33.6	0.12	0.123	0.6	0.2	2.2	0.19	0.014	3.6	2.7	5.8	−0.05	0.513
Spain	13.0	4.9	21.9	0.27	<0.001	14.1	11.0	17.1	−0.13	0.013	0.3	0.0	1.6	0.08	0.066	2.3	1.3	3.4	−0.04	0.281
Sweden	7.5	4.4	11.1	−0.06	0.977	17.4	12.2	30.5	0.19	0.153	2.1	0.9	6.7	0.28	0.021	3.8	2.6	5.5	−0.34	0.000
United Kingdom	13.5	7.7	23.3	−0.09	0.479	16.2	8.8	27.9	0.30	<0.001	0.2	0	1.1	0.12	0.129	4.8	3.1	6.8	−0.16	0.031
Total, women	12.6	6.0	25.1	0.13	0.199	14.7	10.2	25.1	0.16	0.063	0.4	0	1.9	0.11	0.198	3.4	2.0	5.4	−0.13	0.158
p−value	<0.001					<0.001					<0.001					<0.001				
Men, N = 580																				
Denmark	21.1	10.3	32.1	0.28	0.031	31.3	15.1	44.2	0.45	<0.001	0.5	0	1.9	−0.04	0.750	4.5	3.1	6.3	−0.14	0.293
Germany	20.9	10.0	39.9	0.18	0.110	19.6	10.6	42.5	0.31	0.005	0.4	0	3.0	0.31	0.005	4.9	3.0	8.1	0.05	0.660
Greece	9.7	2.5	19.3	0.38	0.002	17.7	13.3	26.2	0.34	0.006	0.1	0	0.4	0.13	0.293	3.7	1.8	7.0	0.04	0.744
Italy	14.0	7.6	33.4	0.47	0.001	16.0	11.2	34.0	0.33	0.020	0.6	0.2	1.4	0.02	0.869	2.3	1.4	3.6	−0.21	0.137
Netherlands	29.6	16.2	46.2	0.06	0.759	14.1	9.7	16.2	−0.06	0.762	1.8	0	5.3	0.16	0.428	4.9	2.1	7.4	−0.12	0.538
Spain	17.0	8.7	35.5	0.20	0.065	14.7	11.4	27.6	0.35	0.001	0.3	0.1	1.6	0.25	0.018	2.9	1.4	4.1	−0.12	0.276
Sweden	11.1	7.0	17.0	−0.06	0.615	20.4	12.7	49.9	0.00	0.995	1.1	0.4	3.9	0.16	0.162	3.2	2.1	4.9	−0.21	0.063
United Kingdom	21.7	11.4	36.9	0.13	0.231	21.9	9.6	50.5	0.48	<0.001	0.2	0	0.9	−0.18	0.082	4.6	3.4	7.2	−0.16	0.121
Total, men	16.0	8.1	30.9	0.19	0.207	18.3	12.3	36.5	0.30	0.204	0.4	0	2.0	0.10	0.264	3.7	2.3	6.2	−0.11	0.328
p-value	<0.001					<0.001					<0.001					<0.001				

Abbreviations: PAEE- physical activity energy expenditure; MVPA- moderate to vigorous physical activity; IQR- interquartile range; r- partial correlation coefficients (r) between domain-specific PA assessed by the RPAQ and objectively measured total PA adjusted for all other domains; PAEE for work was calculated only for participants who reported being employed; p-value for the difference across countries (Kruskal-Wallis test).

### Comparison with Cambridge Index

Spearman’s correlation coefficients between objectively assessed PAEE, MVPA and the Cambridge Index were of similar magnitudes to those for the Short EPIC-PA-questionnaire [30], with pooled estimates of rho = 0.23 (95% CI: 0.18–0.27) and rho = 0.23 (95% CI: 0.19–0.28) for PAEE and time at MVPA, respectively (Supplementary figure 4a), with considerable heterogeneity across countries. The results remained unchanged after using the standard definition of 1MET (Supplementary figure 4b). In addition, we observed a statistically significant trend in objectively measured PAEE and MVPA across the categories of the Cambridge Index (data not shown), which implies that this index ranks participants according to the level of PA.

### Revisiting Occupational Intensity Distribution

Employed participants spent the greatest proportion of time at low intensity levels (Supplementary figures 5 and 6). Intensity distribution during working hours differed substantially by occupational group (p<0.001, multivariate test for means), with more time at higher intensity categories in physically demanding jobs. Similarly, proportion of daily time spent sedentary at work (≤1.5MET) was lower in participants with greater physical demands at work, and ranged from median (IQR) 26% (14%–40%) in heavy manual occupations to 55% (42%–67%) in sedentary occupations. The pattern in unemployed participants resembled that of sedentary workers. Heavy manual workers spent more time in light-intensity PA (1.5–3MET) compared with other occupations.

When applying these intensity distributions from the “training sample” (N = 1282) to the “holdout sample” (N = 641), occupational and total PAEE displayed an increasing trend across occupational groups (Figure 2), with the highest values in heavy manual workers (p<0.001). After applying the empirically-derived intensity distribution to each group, occupational and total PAEE substantially dropped in all occupations (all p<0.001), with the greatest reduction in heavy manual workers. In all employed participants, the revisited median (IQR) for occupational and total RPAQ-derived PAEE were 8.4 (5.6, 13.1) kJ/kg/day (30% lower than in original derivation) and 28.3 (18.8, 43.4) kJ/kg/day (23% lower than in original derivation), respectively. Similarly, median bias (LoA) became materially smaller in manual and heavy manual workers but increased somewhat in sedentary and standing workers (p<0.001 in all groups). The revisited median bias (LoA) for all occupations was −13.4 (−26.0, 0.6) kJ/kg/day, corresponding to 28.8% of median PAEE.

## Discussion

The results of this study suggest that the RPAQ is a valid instrument for the assessment of PAEE, MVPA and SED-time in the adult European population. Although relative validity of questionnaire-derived PAEE and MVPA against objectively measured variables is comparable with previously validated questionnaires, the magnitude of underestimation of PAEE with the RPAQ appears lower compared to other questionnaires.

The observed inter-method correlations for PAEE and MVPA are consistent with the findings based on the Cambridge Index from the Short EPIC-PA-questionnaire [30]. Fair to moderate correlations between questionnaire-derived and objectively assessed PAEE in this study are comparable with previous results using objective criterion methods (accelerometer, HR monitor or combined HR and movement sensor) with correlations around 0.3 [43]–[47]. Prince *et al.* [48] reviewed 173 validation studies and found that the mean (SD) of correlation coefficients between PA-questionnaires and an objective criterion method was 0.37 (0.25), ranging from −0.71 to 0.96. Van Poppel *et al.* [49] systematically reviewed 85 PA-questionnaires for adults and concluded that the methodological quality of studies assessing measurement properties of PA-questionnaires was suboptimal (mainly due to small sample size, inadequate analysis of construct validity or comparison of measures that do not assess the same construct) and that no questionnaire or type of questionnaire is superior to the others. Helmerhorst *et al.* [9] conducted a systematic review of 96 existing and 34 newly developed PA-questionnaires and concluded that majority of the PA-questionnaires had acceptable reliability, but their validity was moderate. Newly developed PA-questionnaires did not appear to perform better than the existing PA-questionnaires with regard to reliability and validity [9]. In addition, the majority of studies reported only correlation coefficients between the questionnaire and the criterion method which precludes comparing absolute validity between studies. Nevertheless, these reviews pointed to the heterogeneity in the differences between self-reported and objectively measured PAEE, with both overestimation and underestimation being probable[48]–[50].

In terms of the comparison with the criterion validity of commonly used surveillance instruments (GPAQ and IPAQ), Spearman’s correlation coefficients between total PA-time from the GPAQ with pedometer- and accelerometer-assessed counts/day in 9 countries [51] were 0.31 and 0.24, respectively, suggesting a similar degree of relative validity as observed for the RPAQ in our study. The corresponding correlation coefficient for sedentary time from GPAQ and accelerometry was 0.40 [51]. For IPAQ (short form), Spearman’s rho varied from −0.12 to 0.57 for total PA, and from 0.07 to 0.61 for sedentary time when compared with corresponding accelerometer-assessed variables [45]. Despite the marked differences between these questionnaires (i.e. questions about time spent in broad intensity levels as opposed to specific activities), validity of estimates appears to be similar. However, unlike IPAQ and GPAQ information, RPAQ information may be summarised in other ways than caloric derivatives evaluated in this article which might be important for specific health outcomes, e.g. activities with an element of weight-bearing may have a beneficial effect on bone density [52], or activities performed in groups present greater opportunity for social interaction [53].

As systematic differences between instruments do not affect the correlation coefficients but may substantially affect agreement, both relative and absolute validity were investigated. The underestimation of PAEE by the RPAQ is consistent with the findings of our previous validation study using doubly labelled water as the criterion [16], but the size of bias in the current larger study is smaller (median(LoA)) for all participants: −4.6 (−46.0, 78.7) kJ/kg/day (−11%), which is equivalent to −77 (−768, 1314) kcal/day for a person with a body weight of the sample mean. Underestimation of PAEE in our study is also in accordance with several other studies that used objective criterion for the validation of PA-questionnaire, but the size of bias of the RPAQ appears to be smaller. For example, the 7-d Physical Activity Recall by Leenders *et al.* [*54*] had a mean bias(LoA) of −156 (−1095, 1306) kcal/day (−20%), the Minnesota Leisure Time Questionnaire [55] a mean bias(LoA) of −313 (−1188, 562) kcal/day (−39%), and the College Alumnus Physical Activity Questionnaire [55] a mean bias of −240 (−1076, 596) kcal/day (−30%).

A consistent finding of positive association of bias with inter-method average in Bland-Altman plots, but inverse association with objectively measured PAEE, MVPA and SED-time suggests that the average was predominantly driven by self-reported parameters that have a different error structure than those obtained by the combined sensor. Therefore, the direction of proportional error should be interpreted with caution.

The observation that SED-time was underestimated by the RPAQ is in line with previous reports [43], [46], [48], [56]. However, the reasons for sex differences in absolute validity of the RPAQ to ascertain MVPA are unclear, but there was evidence of substantial overestimation when the standard definition of 1MET was used to derive objective variables, the definition used in most other studies. The limits of agreement are comparable to the findings of studies which sought to assess absolute validity of PA-questionnaires against an objective method [50].

Furthermore, modest partial correlations were observed between domain-specific PAEE from the RPAQ and objectively measured total PAEE, although the partial correlation for domestic PA was negative. This finding is consistent with previous reports [7], [43]. However, domestic PA assessed by the RPAQ was comprised of mainly sedentary pursuits (TV-viewing at 1MET and computer use at 1.5 MET) and stair climbing (which is generally very short in duration) but did not include household chores, which suggests that PA in this particular domain might have been underestimated or that the assigned energy cost is inaccurate. Given the inability of the criterion method to discriminate the domains of PA, the validity of domain-specific PAEE could not be assessed.

A major strength of this study is its large sample of adult men and women from 10 European countries (representative of the EPIC-cohort with respect to age and sex), which allows examination of country-specific validity. Moreover, HR was individually calibrated, the sensor was worn at two independent time-points for at least 4 consecutive days, and procedures were standardized across the centres, all of which are design features that help improve the precision of the criterion measure. In addition, the objective criterion measure has a different error structure compared to the RPAQ, thus eliminating the possibility of correlated errors which could have occurred had the RPAQ been validated against a PA-log or diary [57]. The RPAQ was administered electronically (except in Sweden), which reduced the time required to distribute the questionnaires and receive answers and eliminated the need for the research teams to enter hand-written responses manually into computer, thus decreasing the possibility of transcription error and significantly reducing research cost. Lastly, combined HR and movement sensing as a criterion measure of PA overcomes some of the weaknesses of HR and accelerometry when used separately; this includes the limited validity of HR monitoring for sedentary behaviour (due to HR being influenced by factors other than PA and therefore being less valid for the assessment of light PA and sedentary behaviour) and inability of accelerometry to capture PAEE during cycling, swimming or upper-body activity [37], [39].

Several limitations need to be considered when interpreting the results of this study. Energy costs estimated from tabulated values [36] do not allow for between-individual variations in PAEE for a given activity [7], [58]. Therefore, a single estimate of energy cost applied to all individuals pursuing a particular activity does not capture different intensities and heterogeneity in mechanical and metabolic efficiency. Prior to calculating RPAQ-derived PAEE, no assumption about within-individual variation in activity intensity was made, i.e. a particular activity is assigned the same intensity for the entire reported duration, yet in reality, it is likely that both within- and between-individual variation in intensity of most activities exist over time. This is especially emphasised in the occupational domain where the hypothesised intensity is used to calculate PAEE for the whole reported work duration (typically >7 hrs/day) notwithstanding its variability during that period. Indeed, when we revisited the intensity distribution assumption during working hours, it led to a considerable decrease in RPAQ-derived PAEE in all occupational categories, and an appreciable reduction in bias in those with the greatest bias (manual and heavy manual workers). Nevertheless, this approach seems to slightly increase the bias in sedentary occupations, which were the most prevalent in this sample, thus leading to a worsening of the sample bias. The analysis is limited by a low number of participants with physically demanding occupations, and therefore the utility of the empirically-derived intensity distribution should be tested in a larger sample to gain a better insight into its impact on questionnaire-derived PAEE and bias. Future research should also investigate the effect of this approach on the associations between occupational PA and health outcomes.

An intrinsic limitation of objective PA-monitoring is that it typically provides only a snap-shot of an individual’s habitual PA (4–5 days), whereas the reference period of the RPAQ was past 4 weeks. Despite measuring PA objectively at two visits and capturing a range of PA-patterns, differences in PA between weekend days and weekdays might have been ascertained in a more detail over one entire week of monitor wear. Similarly, seasonal variation in PA might not have been reliably captured by only two assessments, a phenomenon which applies to a single administration of the RPAQ as well [59].

In conclusion, the relative and absolute validities of the RPAQ in estimating PAEE and MVPA are consistent with the results of previous validation studies and the limitations (e.g. bias and weak correlations) need to be considered when interpreting RPAQ-data. For example, population estimates of PAEE would be valid, whereas using the tool to address aetiological questions would result in an attenuation of risk estimates between activity and disease. Nevertheless, the electronic RPAQ is a convenient tool and can be used with reasonable confidence in large-scale epidemiological studies in European countries to compare population estimates of total and domain-specific PA as well as other summary measures of PA, and to examine associations between PA and health outcomes.

## Supporting Information

Figure S1 **Bland-Altman plots of physical activity energy expentiture (kJ/kg/day), time in moderate-to-vigorous physical activity (min/day) and sedentary time (h/day) from RPAQ and combined sensing stratified by sex using individualised definition of 1MET; solid line represents median bias, and dashed lines denote limits of agreement.**
(TIFF)

Figure S2 **Bland-Altman plots of time in moderate-to-vigorous physical activity (min/day) and sedentary time (h/day) from RPAQ and combined sensing stratified by sex using standard definition of 1MET = 3.5 ml O2/kg/min (1343 women and 540 men); solid line represents median bias, and dashed lines denote limits of agreement.**
(TIFF)

Figure S3 **Spearman’s correlation coefficients for the associations of MVPA and sedentary time assessed by the RPAQ with objectively measured corresponding variables by country and sex using standard definition of 1MET = 3.5 ml O2/kg/min (1343 women and 540 men).**
(TIFF)

Figure S4 **Spearman's correlation coefficients for the associations of objectively assessed PAEE and time at MVPA with the Cambridge Index in the RPAQ validation study cohort (N = 1923, 1343 women and 540 men).**
(TIFF)

Figure S5 **Intensity distribution during working hours from Monday to Friday by occupational group Index in the RPAQ validation study cohort (N = 1923, 1343 women and 540 men) using individualised definition of 1 MET.** Inserts of each graph show zoomed view of intensity distribution in the MVPA (>3 METs) zone. All values have been normalised to bin size 0.25 METs. Data are median (IQR).
(TIFF)

Figure S6 **Intensity distribution during working hours from Monday to Friday by occupational group Index in the RPAQ validation study cohort (N = 1923, 1343 women and 540 men) using standard definition of 1MET = 3.5 ml O2/kg/min.** Inserts of each graph show zoomed view of intensity distribution in the MVPA (>3 METs) zone. All values have been normalised to bin size 0.25 METs. Data are median (IQR).
(TIFF)

Table S1 **Time spent in moderate to vigorous physical activity (min/day) as assessed by the Recent Physical Activity Questionnaire and combined movement sensor and heart rate monitor, N = 1923.** Abbreviations: IQR- interquartile range; LOA- limits of agreement; range of bias includes the values between 2.5^th^ and 97.5^th^ percentile; Acc+HR- combined accelerometer and heart rate monitor; Monitor data in this analysis was processed using standard definition of 1 MET (3.5 ml O2/kg/min). *p<0.05, **p<0.01, ***p<0.001 for bias.
(DOC)

Table S2 **Time spent sedentary (h/day) as assessed by the Recent Physical Activity Questionnaire and combined movement sensor and heart rate monitor, N = 1923.** Abbreviations: IQR- interquartile range; LOA- limits of agreement; range of bias includes the values between 2.5^th^ and 97.5^th^ percentile; Acc+HR- combined accelerometer and heart rate monitor; Monitor data in this analysis was processed using standard definition of 1 MET (3.5 ml O2/kg/min). *p<0.05, **p<0.01, ***p<0.001 for bias.
(DOC)
