# Correction: Validity of Electronically Administered Recent Physical Activity Questionnaire (RPAQ) in Ten European Countries

**DOI:** 10.1371/journal.pone.0114103

**Published:** 2014-11-26

**Authors:** The PLOS ONE Staff

There are multiple errors in the “Results” subsection of the Abstract. The correct paragraph is: RPAQ significantly underestimated PAEE in women [median(IQR): 34.9 (22.3, 52.8) vs. 40.6 (32.4, 50.9) kJ/kg/day, 95%LoA: -44.4, 66.1 kJ/kg/day) and overestimated PAEE in men [45.9 (30.6, 71.1) vs. 45.5 (34.1, 57.6) kJ/kg/day, 95%LoA: -44.8, 102.6 kJ/kg/day]. Using individualised definition of 1MET, RPAQ significantly underestimated MVPA in women [median(IQR): 63.7 (30.5, 126.9) vs. 73.6 (47.8, 107.2) min/day, 95%LoA: -127.4, 311.9 min/day] and overestimated MVPA in men [90.0 (42.3, 188.6) vs. 83.3 (55.1, 125.0) min/day, 95%LoA: -134.8, 427.3 min/day]. Correlations (95%CI) between subjective and objective estimates were statistically significant [PAEE: women, rho = 0.20 (0.15-0.26); men, rho = 0.37 (0.30-0.44); MVPA: women, rho = 0.18 (0.13-0.24); men, rho = 0.31 (0.24-0.38)]. When using non-individualised definition of 1MET (3.5 mlO_2_/kg/min), MVPA was substantially overestimated (16 min/day, and 32 min/day in women and men, respectively). Revisiting occupational intensity assumptions in questionnaire estimation algorithms with occupational group-level empirical distributions reduced median PAEE-bias in manual (38.8 kJ/kg/day vs. 6.8 kJ/kg/day, p<0.001) and heavy manual workers (63.6 vs. -2.8 kJ/kg/day, p<0.001) in an independent hold-out sample.

There is an error in the first sentence of the Introduction. The correct sentence is: Epidemiological studies have demonstrated that physical inactivity is an important determinant of numerous chronic diseases, including type 2 diabetes, obesity, cardiovascular disease and certain types of cancer[1]–[3].

There is an error in the first sentence of the second paragraph of the Introduction. The correct sentence is: Physical activity (PA) is a complex behaviour that is difficult to assess accurately in free-living individuals [5].

There are multiple errors in the first three sentences of the “Absolute validity” portion of the “Physical activity energy expenditure” subsection of the Results. The correct sentences are: The RPAQ underestimated PAEE in women, with a significant median bias (LoA) of -6.0 (-44.4, 66.1) kJ/kg/day, corresponding to -15% of median PAEE (Table 2). In men, median bias (LoA) was positive at 2.5 (-44.8, 102.6) kJ/kg/day (5.5% of objective median). Median bias (LoA) for all participants was -3.6 (-44.8, 79.1) kJ/kg/day (-7.7%), which was significantly different from 0.

There is an error in the last sentence of the “Relative validity” portion of the “Physical activity energy expenditure” subsection of the Results. The correct sentence is: The pooled estimate in men was substantially (p = 0.003) greater than that in women, rho = 0.37 (95% CI: 0.30 to 0.44) with moderate heterogeneity by country (*I^2^*  = 49.5%, p = 0.054).

There are multiple errors in the first two sentences of the “Absolute validity” portion of the “Time in moderate-to-vigorous physical activity” subsection of Results. The correct sentences are: When using individualised RMR to define objective MVPA, the RPAQ significantly underestimated MVPA (Table 3) in women with median bias (LoA) -7.5 (-127.4, 311.9) min/day (-10.2%), and significantly overestimated in men, with median bias (LoA) 12.1 (-134.8, 427.3) min/day (14.5%). There was a material underestimation in both sexes combined, with median bias (LoA) -3.0 (-131.1, 363.7) min/day (-4.0%).

There are multiple errors in the “Relative validity” portion of the “Time in moderate-to-vigorous physical activity” subsection of Results. The correct paragraph is: Inter-method correlation for MVPA (Figure 1) was slightly weaker than that observed for total PAEE and greater for men than women, p = 0.003 (rho = 0.18, 95% CI: 0.13 to 0.24; *I^2^*  =  64.1%, p = 0.003 for women and rho = 0.31, 95% CI: 0.24 to 0.38; *I^2^*  =  71.8%, p = 0.001 for men). Comparative pooled correlation coefficients using the standard definition of 1MET were rho = 0.17, 95% CI: 0.11 to 0.22; *I^2^*  =  74.3%, p<0.001 in women, and rho = 0.26, 95% CI: 0.19 to 0.34; *I^2^*  =  74.8%, p<0.001 in men (Supplementary figure 3); p = 0.007 for the difference in rho between the sexes.

There is an error in the last sentence of the “Relative validity” portion of the “Sedentary time” subsection of the Results. The correct sentence is: When using the standard definition of 1MET, pooled estimate was rho = 0.19 (95% CI: 0.14 to 0.24), *I^2^*  = 42.8%, p = 0.072 in women and rho = 0.22 (95% CI: 0.13 to 0.30), *I^2^*  = 0%, p = 0.949 in men (Supplementary figure 3).

There is an error in the second sentence of the second paragraph of the “Domain-specific PAEE from the RPAQ and total objectively assessed PAEE” subsection of the Results. The correct sentence is: After adjustment for all other domains, correlation coefficients varied by country, and overall there was a weak positive correlation for the occupational domain (women: r = 0.16; men: r = 0.30), leisure time PA (women: r = 0.14; men: r = 0.17) and commuting PA (women: r = 0.11; men: r = 0.10) but a weak negative correlation for PAEE in the home domain (women; r = -0.13; men: r = -0.11).

There are multiple errors in the second paragraph of the “Revisiting occupational intensity distribution” subsection of the Results. The correct paragraph is: When applying these intensity distributions from the “training sample” (N = 1282) to the “holdout sample” (N = 641), occupational and total PAEE displayed an increasing trend across occupational groups (Figure 2), with the highest values in heavy manual workers (p<0.001). After applying the empirically-derived intensity distribution to each group, occupational and total PAEE substantially dropped in all occupations (all p<0.001), with the greatest reduction in heavy manual workers. In all employed participants, the revisited median (IQR) for occupational and total RPAQ-derived PAEE were 8.4 (5.6, 13.1) kJ/kg/day (30% lower than in original derivation) and 30.6 (20.3, 45.2) kJ/kg/day (23% lower than in original derivation), respectively. Similarly, median bias became materially smaller in manual (38.8 kJ/kg/day vs. 6.8 kJ/kg/day, p<0.001) and heavy manual workers (63.6 vs. -2.8 kJ/kg/day, p<0.001) in the hold-out sample, but increased somewhat in sedentary and standing workers (p<0.001 in all groups). The revisited median bias (LoA) for all occupations was -10.5 (-51.0, 56.1) kJ/kg/day, corresponding to 25.9% of median PAEE.

There are multiple errors in the third to last sentence of the third paragraph of the Discussion. The correct sentence is: The underestimation of PAEE by the RPAQ is consistent with the findings of our previous validation study using doubly labelled water as the criterion^16^, but the size of bias in the current larger study is smaller [median(LoA)] for all participants: -3.6 (-44.8, 79.1) kJ/kg/day (-7.7%), which is equivalent to -62 (-771, 1361) kcal/day for a person with a body weight of the sample mean.

There are multiple errors in Table 2. Please see the corrected Table 2 here.

**Table 2:** Physical activity energy expenditure (kJ/kg/day) as assessed by the Recent Physical Activity Questionnaire and combined movement sensor and heart rate monitor, N = 1923.

	RPAQ					Acc+HR					Inter-method difference			
	Mean	SD	Median	IQR		Mean	SD	Median	IQR		Mean bias	Median bias	LoA	
Women, N = 1343														
Denmark	53.8	35.6	47.4	27.0	68.4	39.0	11.8	38.0	30.2	47.2	14.8	6.0***	-28.9	104.1
France	34.2	20.9	29.7	19.6	43.8	38.2	11.9	37.5	30.8	45.4	-4.0	-8.9*	-33.8	50.8
Germany	46.2	35.2	36.7	24.5	54.8	40.7	14.3	38.7	30.0	49.7	5.5	-1.0	-30.0	64.0
Greece	28.6	21.6	21.9	13.6	39.2	38.8	14.1	38.2	29.7	48.1	-10.2	-14.0***	-43.0	36.5
Italy	31.5	18.8	25.7	18.8	39.2	46.3	13.6	44.3	36.8	55.5	-14.8	-16.6	-50.6	23.4
Netherlands	57.2	26.1	51.5	40.4	72.4	46.5	17.6	43.6	35.4	54.9	10.7	8.6***	-44.9	81.2
Norway	40.8	25.2	35.1	23.1	53.0	45.1	14.5	42.7	35.1	53.7	-4.3	-7.4*	-48.7	58.7
Spain	32.9	17.3	29.6	21.1	42.6	47.9	13.5	46.0	38.6	57.1	-15.0	-15.3***	-51.0	30.9
Sweden	47.5	33.9	41.2	26.4	57.3	42.9	14.6	41.0	32.1	51.7	4.6	-1.3	-32.3	82.3
United Kingdom	36.3	22.3	29.2	19.1	49.9	35.3	11.6	33.5	26.4	44.1	1.0	-3.3	-36.8	54.6
Total, women	41.1	27.6	34.9	22.3	52.8	42.4	14.5	40.6	32.4	50.9	-1.3	-6.0*	-44.4	66.1
Men, N = 580														
Denmark	71.2	61.0	52.8	38.8	80.6	43.1	17.4	41.5	32.4	51.8	28.1	13.1***	-26.5	232.9
Germany	62.3	39.4	51.0	35.8	78.1	41.6	13.9	41.0	31.3	49.4	20.7	14.0***	-27.9	94.6
Greece	40.5	30.0	30.6	22.4	45.7	44.2	19.3	43.8	29.4	54.7	-4.7	-7.3	-45.7	63.9
Italy	44.5	25.9	42.0	28.2	54.4	51.2	15.8	48.7	41.5	60.1	-6.7	-12.1*	-37.4	44.7
Netherlands	56.8	24.4	54.2	38.5	66.3	55.2	15.1	54.0	44.6	63.7	1.6	0.7	-63.7	74.3
Spain	50.2	34.8	44.7	28.5	59.4	51.1	19.5	48.6	38.2	62.5	-0.5	-5.1	-47.8	55.1
Sweden	62.6	35.7	54.0	37.0	80.2	55.3	18.8	52.4	41.3	67.0	7.6	1.5	-55.6	113.1
United Kingdom	61.0	72.7	47.5	25.9	74.3	40.0	16.2	36.6	29.3	48.9	21.0	7.9*	-39.4	105.5
Total, men	56.9	46.7	45.9	30.6	71.1	47.1	18.2	45.5	34.1	57.6	9.8	2.5***	-44.8	102.6
Total, both sexes	45.8	35.2	38.3	24.1	57.9	43.6	15.8	46.5	32.7	53.3	2.0	-3.6*	-44.8	79.1

IQR- interquartile range; SD- standard deviation; LoA- 95% limits of agreement; range of bias includes the values between 2.5^th^ and 97.5^th^ percentile;

Acc+HR- combined accelerometer and heart rate monitor

*p<0.05, **p<0.01, ***p<0.001 for bias

There are multiple errors in Table 3. Please see the corrected Table 3 here.

**Table 3:** Time spent in moderate and vigorous physical activity (min/day) as assessed by the Recent Physical Activity Questionnaire and combined movement sensor and heart rate monitor, N = 1923.

	RPAQ					Acc+HR					Inter-method difference			
	Mean	SD	Median	IQR		Mean	SD	Median	IQR		Mean bias	Median bias	LoA	
Women, N = 1343														
Denmark	107.4	100.0	71.8	46.1	120.0	72.5	37.4	63.5	45.2	95.5	34.8	3.1***	-90.3	322.0
France	71.9	84.1	48.6	23.4	86.8	71.6	37.6	65.4	46.3	88.8	0.3	-17.1	-122.0	262.9
Germany	106.5	97.4	74.0	41.9	133.9	80.1	46.1	73.0	46.1	100.3	26.5	7.1**	-117.0	294.2
Greece	69.2	92.5	41.4	19.8	72.6	70.5	40.6	68.1	42.9	92.3	-1.4	-15.1	-102.0	275.0
Italy	78.2	91.5	41.9	18.5	103.9	80.4	52.3	72.5	46.0	101.0	-2.2	-21.5	-137.9	264.4
Netherlands	169.1	105.1	146.3	99.1	205.7	97.6	57.8	90.0	57.3	124.0	71.5	61.2***	-101.2	368.2
Norway	100.8	118.3	53.6	24.4	114.8	91.0	53.8	79.2	54.8	119.2	9.8	-22.5	-130.5	348.8
Spain	70.4	59.3	56.4	26.2	99.9	96.4	49.5	89.2	58.4	125.6	-26.0	-30.4**	-143.3	163.3
Sweden	99.7	108.7	62.1	34.4	112.0	91.2	44.9	85.1	55.1	120.8	8.6	-22.7	-136.2	376.0
United Kingdom	86.8	88.0	58.0	35.2	101.7	60.5	40.1	50.9	29.2	84.1	26.3	5.5**	-110.3	271.7
Total, women	98.3	101.2	63.7	30.5	126.9	81.9	48.6	73.6	47.8	107.2	16.3	-7.5***	-127.4	311.9
Men, N = 580														
Denmark	155.8	144.5	100.7	45.0	210.1	82.3	53.3	75.0	42.3	112.4	73.5	26.9***	-108.7	399.9
Germany	175.5	166.8	112.5	49.8	257.6	83.8	43.1	77.5	50.5	109.8	91.7	41.1***	-98.1	427.3
Greece	84.4	121.0	39.7	10.7	98.0	96.4	89.1	82.5	38.3	137.2	-12.0	-26.5	-132.1	285.4
Italy	101.3	113.3	53.4	38.0	120.3	86.1	42.2	81.0	56.4	113.5	15.1	-13.6	-124.1	385.7
Netherlands	116.1	88.2	95.9	52.2	148.1	110.2	47.3	101.8	80.9	138.5	5.9	-4.6	-195.3	283.3
Spain	120.1	152.4	74.3	35.6	140.0	101.3	59.6	92.7	62.8	139.0	18.8	-8.0	-146.1	334.3
Sweden	195.0	168.0	122.0	63.7	307.8	128.2	70.2	111.6	73.7	166.3	66.8	21.5	-206.5	505.3
United Kingdom	184.5	168.1	122.2	49.8	293.2	79.4	62.2	64.1	41.5	115.3	105.1	51.7***	-83.5	534.5
Total, men	148.8	154.3	90.0	42.3	188.6	95.9	63.4	83.3	55.1	125.0	52.9	12.1***	-134.8	427.3
Total, both sexes	107.0	121.4	63.7	28.2	135.5	85.4	53.4	75.7	48.2	111.4	21.5	-3.0***	-131.6	363.7

IQR- interquartile range; SD- standard deviation; LoA- 95% limits of agreement; range of bias includes the values between 2.5^th^ and 97.5^th^ percentile; Acc+HR- combined accelerometer and heart rate monitor

*p<0.05, **p<0.01, ***p<0.001 for bias

There are multiple errors in Table 5. Please see the corrected Table 5 here.

**Table 5:** Domain-specific energy expenditure from the RPAQ and partial correlation with objectively assessed physical activity energy expenditure adjusted for all other domains (580 men and 1343 women).

	PAEE for leisure (kJ/kg/day)					PAEE at work (kJ/kg/day)					PAEE for commuting (kJ/kg/day)					PAEE at home (kJ/kg/day)				
	Median	IQR		r	p-value for r	Median	IQR		r	p-value for r	Median	IQR		r	p-value for r	Median	IQR		r	p-value for r
Women, N = 1343																				
Denmark	15.3	10.8	24.5	0.12	0.010	15.9	12.5	32.0	0.06	<0.001	0.9	0	2.8	0.12	0.375	4.1	2.5	5.8	-0.25	0.005
France	9.3	5.4	18.3	0.11	0.138	13.9	9.2	20.4	0.21	0.006	0.4	0	1.3	0.10	0.175	3.5	2.1	5.7	-0.24	0.002
Germany	14.8	8.2	29.4	0.22	0.004	16.3	11.5	23.2	0.19	0.002	0.6	0.1	1.7	0.11	0.008	3.2	1.9	5.0	-0.23	0.085
Greece	8.5	3.6	14.6	0.18	<0.001	15.0	10.1	29.2	0.31	<0.001	0.0	0	0.4	0.01	0.210	3.0	1.7	4.7	-0.13	0.508
Italy	7.9	3.5	19.0	0.23	<0.001	13.7	10.9	20.7	0.31	<0.001	0.4	0	1.7	-0.01	0.960	2.0	1.2	3.6	0.28	0.043
Netherlands	37.9	26.4	58.1	-0.03	0.549	8.2	3.6	14.6	0.08	0.254	0.5	0	4.3	0.13	0.031	3.8	2.4	5.7	-0.17	0.043
Norway	9.6	4.4	16.8	0.30	<0.001	15.9	11.6	33.6	0.12	0.123	0.6	0.2	2.2	0.19	0.014	3.6	2.7	5.8	-0.05	0.513
Spain	13.0	4.9	21.9	0.27	<0.001	14.1	11.0	17.1	-0.13	0.013	0.3	0.0	1.6	0.08	0.066	2.3	1.3	3.4	-0.04	0.281
Sweden	12.5	7.8	19.9	-0.05	0.725	17.4	12.2	30.5	0.19	0.153	2.1	0.9	6.7	0.28	0.021	3.8	2.6	5.5	-0.34	0.000
United Kingdom	13.5	7.7	23.3	-0.09	0.479	16.2	8.8	27.9	0.30	<0.001	0.2	0	1.1	0.12	0.129	4.8	3.1	6.8	-0.16	0.031
Total, women	13.2	6.5	25.3	0.14	0.725	14.7	10.2	25.1	0.16	0.063	0.4	0	1.9	0.11	0.198	3.4	2.0	5.4	-0.13	0.158
p-value	<0.001					<0.001					<0.001					<0.001				
Men, N = 580																				
Denmark	21.1	10.3	32.1	0.28	0.031	31.3	15.1	44.2	0.45	<0.001	0.5	0	1.9	-0.04	0.750	4.5	3.1	6.3	-0.14	0.293
Germany	20.9	10.0	39.9	0.18	0.110	19.6	10.6	42.5	0.31	0.005	0.4	0	3.0	0.31	0.005	4.9	3.0	8.1	0.05	0.660
Greece	9.7	2.5	19.3	0.38	0.002	17.7	13.3	26.2	0.34	0.006	0.1	0	0.4	0.13	0.293	3.7	1.8	7.0	0.04	0.744
Italy	14.0	7.6	33.4	0.47	0.001	16.0	11.2	34.0	0.33	0.020	0.6	0.2	1.4	0.02	0.869	2.3	1.4	3.6	-0.21	0.137
Netherlands	29.6	16.2	46.2	0.06	0.759	14.1	9.7	16.2	-0.06	0.762	1.8	0	5.3	0.16	0.428	4.9	2.1	7.4	-0.12	0.538
Spain	17.0	8.7	35.5	0.20	0.065	14.7	11.4	27.6	0.35	0.001	0.3	0.1	1.6	0.25	0.018	2.9	1.4	4.1	-0.12	0.276
Sweden	20.4	13.1	29.1	-0.04	0.615	20.4	12.7	49.9	0.00	0.995	1.1	0.4	3.9	0.16	0.162	3.2	2.1	4.9	-0.21	0.063
United Kingdom	21.7	11.4	36.9	0.13	0.231	21.9	9.6	50.5	0.48	<0.001	0.2	0	0.9	-0.18	0.082	4.6	3.4	7.2	-0.16	0.121
Total, men	18.4	9.4	33.2	0.17	0.350	18.3	12.3	36.5	0.30	0.204	0.4	0	2.0	0.10	0.264	3.7	2.3	6.2	-0.11	0.328
p-value	<0.001					<0.001					<0.001					<0.001				

Abbreviations: PAEE- physical activity energy expenditure; MVPA- moderate to vigorous physical activity; IQR- interquartile range; r- partial correlation coefficients (r) between domain-specific PA assessed by the RPAQ and objectively measured total PA adjusted for all other domains; PAEE for work was calculated only for participants who reported being employed; p-value for the difference across countries (Kruskal-Wallis test)

There are multiple errors in Figure 1. Please see the corrected Figure 1 here.

**Figure 1:**
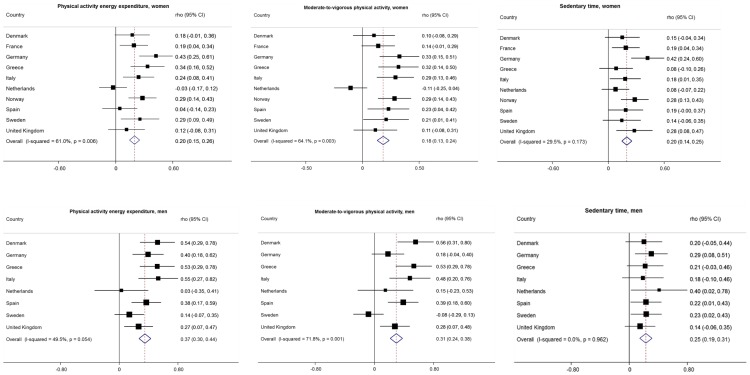
Spearman’s correlation coefficients for the associations of physical activity energy expenditure, moderate-to-vigorous physical activity and sedentary time assessed by the RPAQ with objectively measured corresponding variables by country and sex in 1343 women and 540 men. There are errors in Table S1 and Figures S1-S6 of the Supporting Information. Please view the corrected files below.

## Supporting Information

Table S1 **Time spent in moderate to vigorous physical activity (min/day) as assessed by the Recent Physical Activity Questionnaire and combined movement sensor and heart rate monitor, N = 1923.**
(DOCX)

Figure S1 **Bland-Altman plots of physical activity energy expentiture (kJ/kg/day), time in moderate-to-vigorous physical activity (min/day) and sedentary time (h/day) from RPAQ and combined sensing stratified by sex using individualised definition of 1MET; solid line represents median bias, and dashed lines denote limits of agreement.**
(TIF)

Figure S2 **Bland-Altman plots of time at moderate-to-vigorous physical activity (min/day) and sedentary time (h/day) from RPAQ and combined sensing stratified by sex using standard definition of 1MET  = 3.5 ml O2/kg/min (1343 women and 540 men); solid line represents median bias, and dashed lines denote limits of agreement.**
(TIF)

Figure S3 **Spearman’s correlation coefficients for the associations of time at moderate-to-vigorous physical activity and sedentary time assessed by the RPAQ with objectively measured corresponding variables by country and sex using standard definition of 1MET  = 3.5 ml O2/kg/min (1343 women and 540 men).**
(TIF)

Figure S4 **Spearman's correlation coefficients for the associations of objectively assessed physical activity energy expenditure and time at moderate-to-vigorous physical activity with the Cambridge Index in the RPAQ validation study cohort (N = 1923, 1343 women and 540 men).**
(TIF)

Figure S5 **Intensity distribution during working hours from Monday to Friday by occupational group in the RPAQ validation study cohort (N = 1923, 1343 women and 540 men) using individualised definition of 1 MET.** Inserts of each graph show zoomed view of intensity distribution in the MVPA (>3 METs) zone. All values have been normalised to bin size 0.25 METs. Data are median (IQR).
(TIF)

Figure S6 **Intensity distribution during working hours from Monday to Friday by occupational group in the RPAQ validation study cohort (N = 1923, 1343 women and 540 men) using standard definition of 1MET  = 3.5 ml O2/kg/min.** Inserts of each graph show zoomed view of intensity distribution in the MVPA (>3 METs) zone. All values have been normalised to bin size 0.25 METs. Data are median (IQR).
(TIF)
