# Supplementary material for: Correction: Validity of Electronically Administered Recent Physical Activity Questionnaire (RPAQ) in Ten European Countries
Source: PLoS One. 2014 Nov 26;9(11):e114103. doi: 10.1371/journal.pone.0114103 (PMC4245245; doi:10.1371/journal.pone.0114103)
Supplement: Table S1 — Time spent in moderate to vigorous physical activity (min/day) as assessed by the Recent Physical Activity Questionnaire and combined movement sensor and heart rate monitor, N = 1923. (DOCX) [file pone.0114103.s001.docx]

**Supplementary table 1. Time spent in moderate to vigorous physical activity (min/day) as assessed by the Recent Physical Activity Questionnaire and combined movement sensor and heart rate monitor, N=1923**

Abbreviations: IQR- interquartile range; LOA- limits of agreement; range of bias includes the values between 2.5^th^ and 97.5^th^ percentile; Acc+HR- combined accelerometer and heart rate monitor;

Monitor data in this analysis was processed using standard definition of 1 MET (3.5 ml O2/kg/min)

*p<0.05, **p<0.01, ***p<0.001 for bias
