# Supplementary figures and images for: Correction: Validity of Electronically Administered Recent Physical Activity Questionnaire (RPAQ) in Ten European Countries
Source: PLoS One. 2014 Nov 26;9(11):e114103. doi: 10.1371/journal.pone.0114103 (PMC4245245; doi:10.1371/journal.pone.0114103)

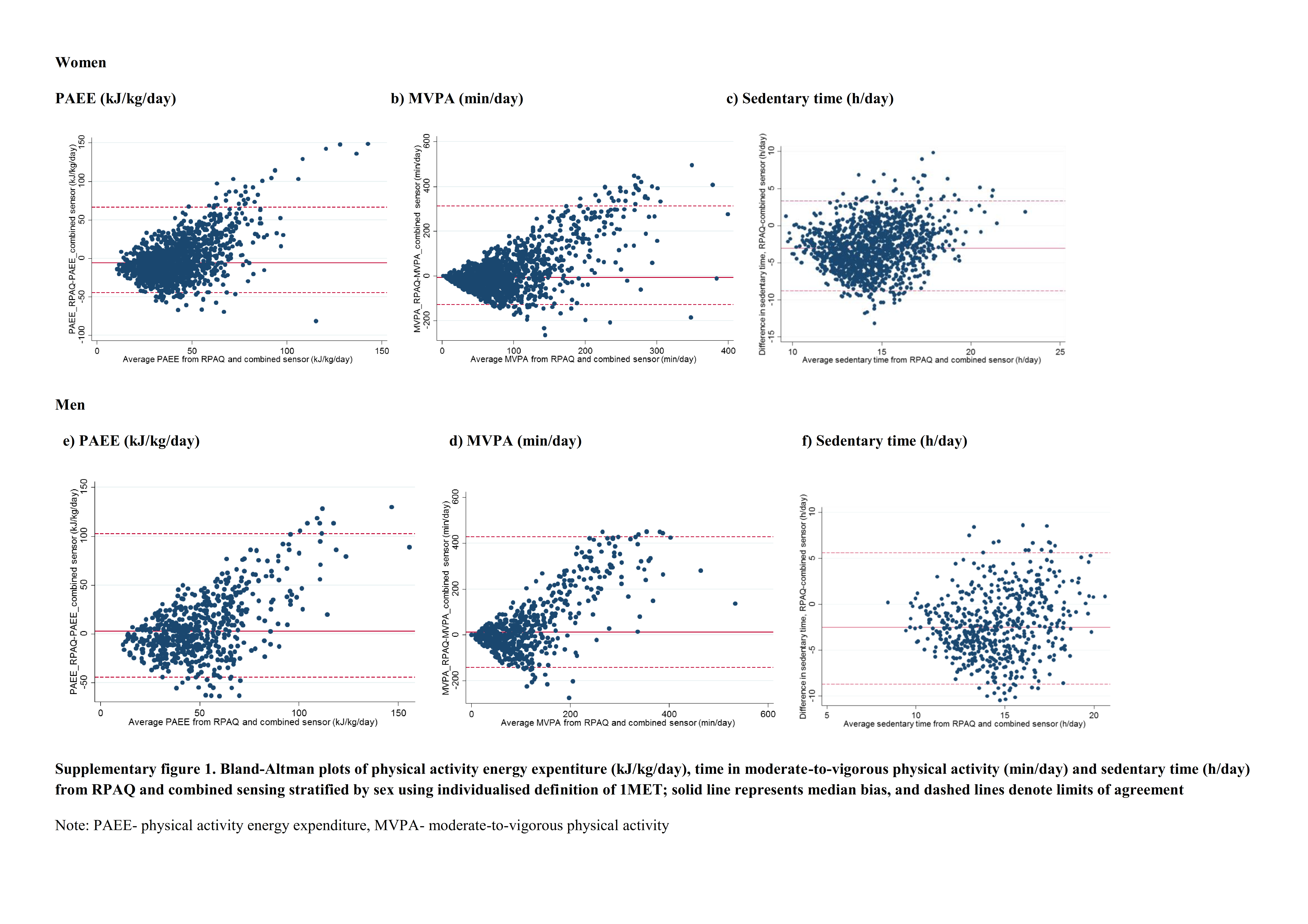

Supplement: Figure S1 — Bland-Altman plots of physical activity energy expentiture (kJ/kg/day), time in moderate-to-vigorous physical activity (min/day) and sedentary time (h/day) from RPAQ and combined sensing stratified by sex using individualised definition of 1MET; solid line represents median bias, and dashed lines denote limits of agreement. (TIF) [file pone.0114103.s002.tif]

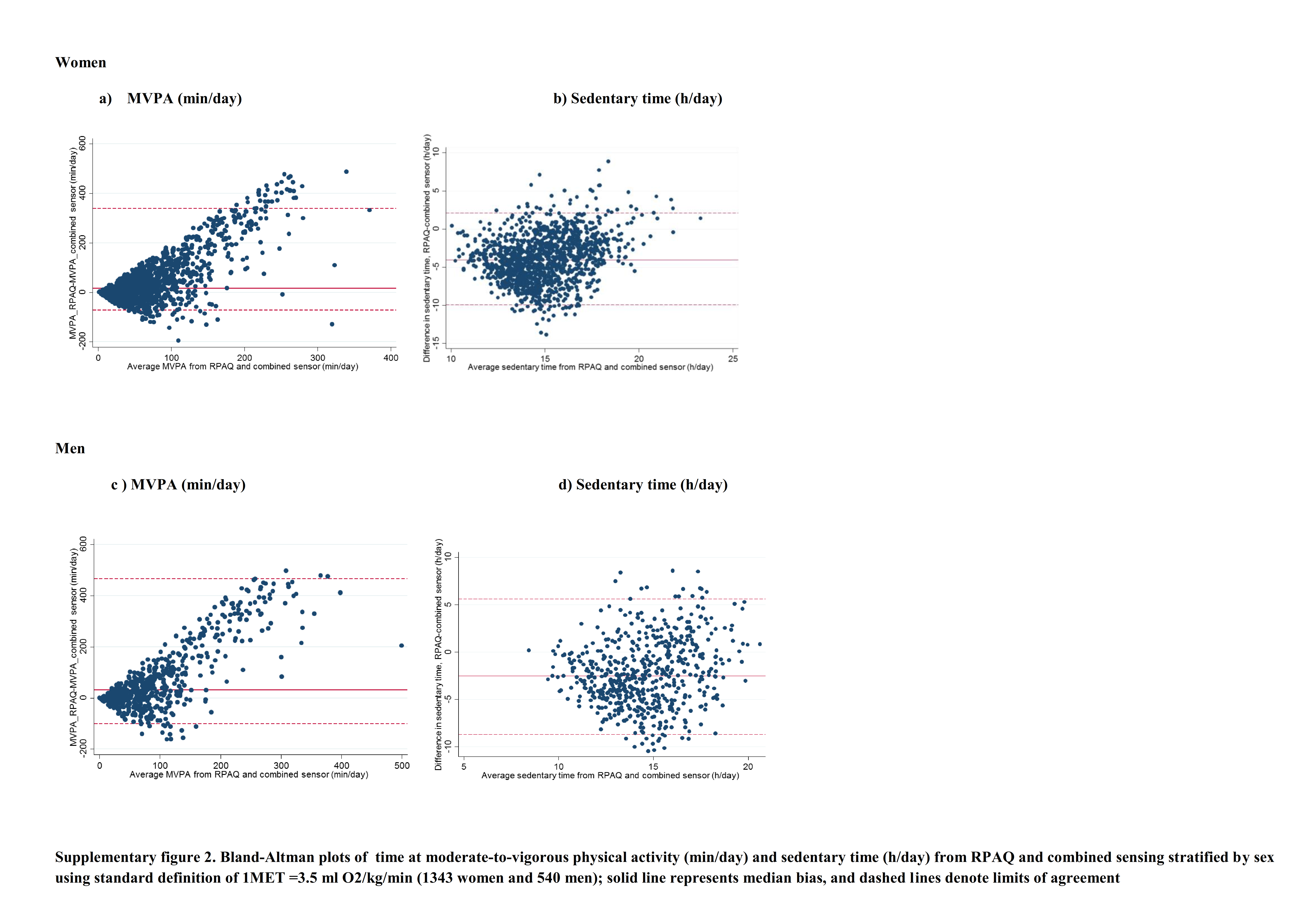

Supplement: Figure S2 — Bland-Altman plots of time at moderate-to-vigorous physical activity (min/day) and sedentary time (h/day) from RPAQ and combined sensing stratified by sex using standard definition of 1MET = 3.5 ml O2/kg/min (1343 women and 540 men); solid line represents median bias, and dashed lines denote limits of agreement. (TIF) [file pone.0114103.s003.tif]

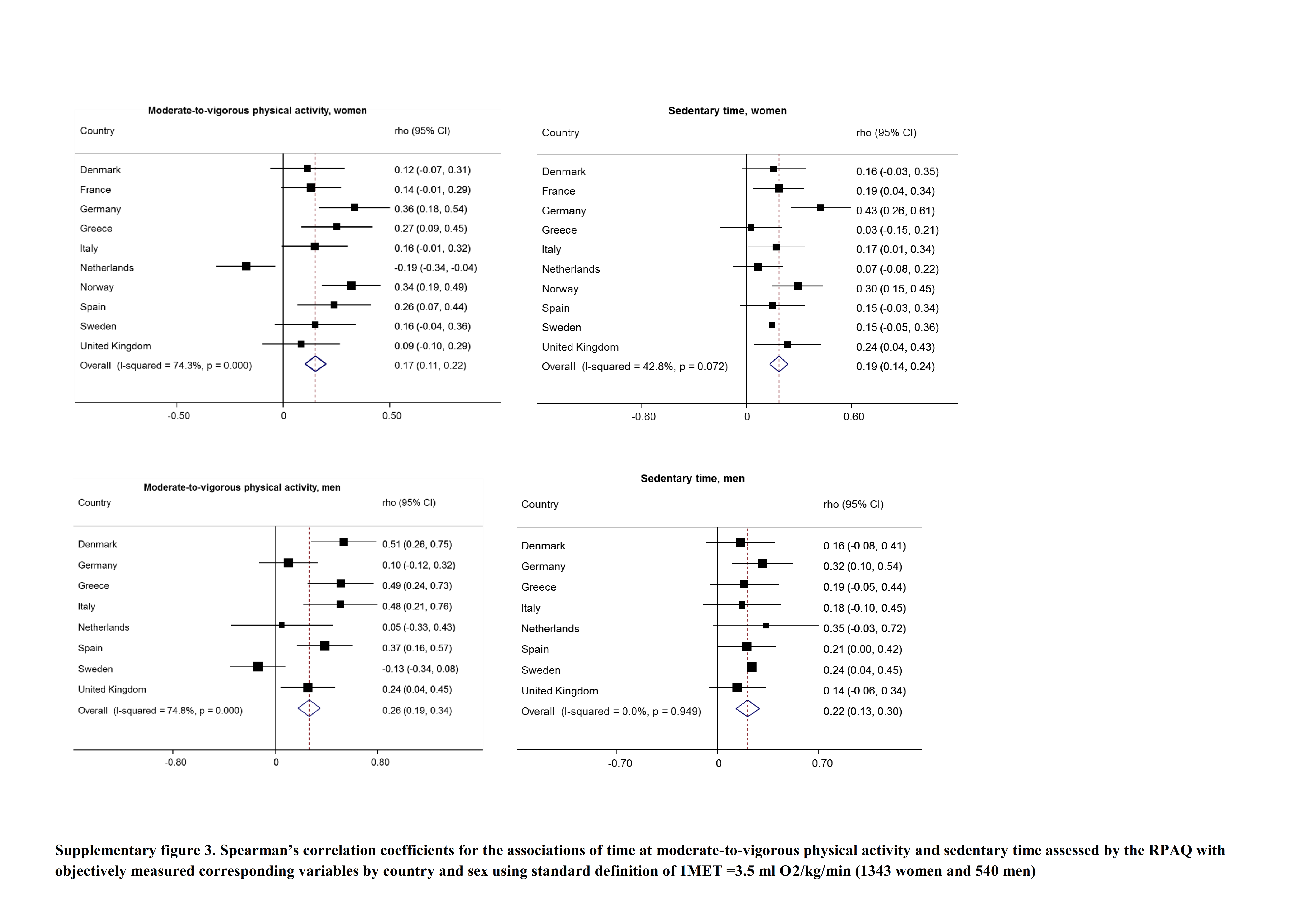

Supplement: Figure S3 — Spearman’s correlation coefficients for the associations of time at moderate-to-vigorous physical activity and sedentary time assessed by the RPAQ with objectively measured corresponding variables by country and sex using standard definition of 1MET = 3.5 ml O2/kg/min (1343 women and 540 men). (TIF) [file pone.0114103.s004.tif]

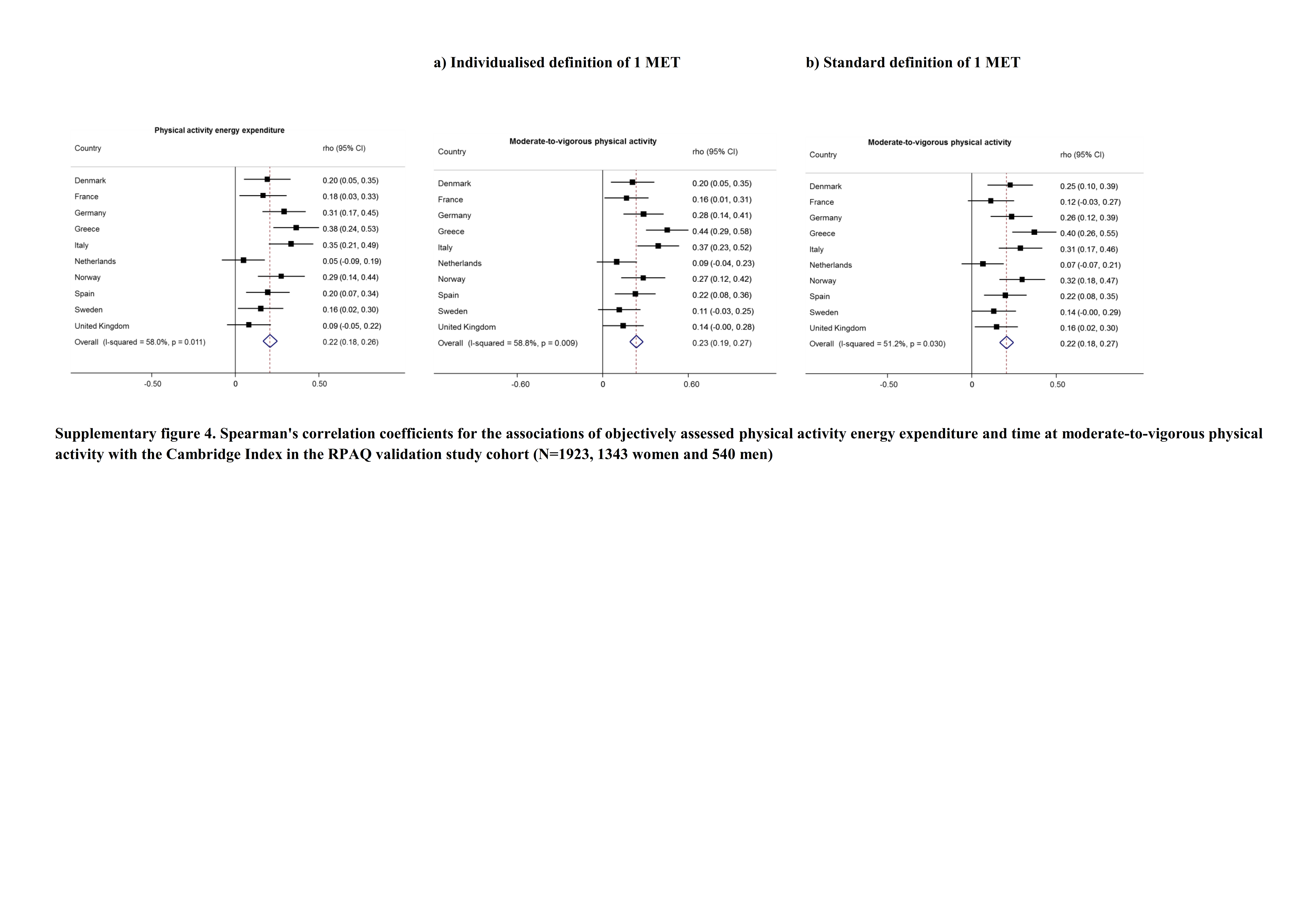

Supplement: Figure S4 — Spearman's correlation coefficients for the associations of objectively assessed physical activity energy expenditure and time at moderate-to-vigorous physical activity with the Cambridge Index in the RPAQ validation study cohort (N = 1923, 1343 women and 540 men). (TIF) [file pone.0114103.s005.tif]

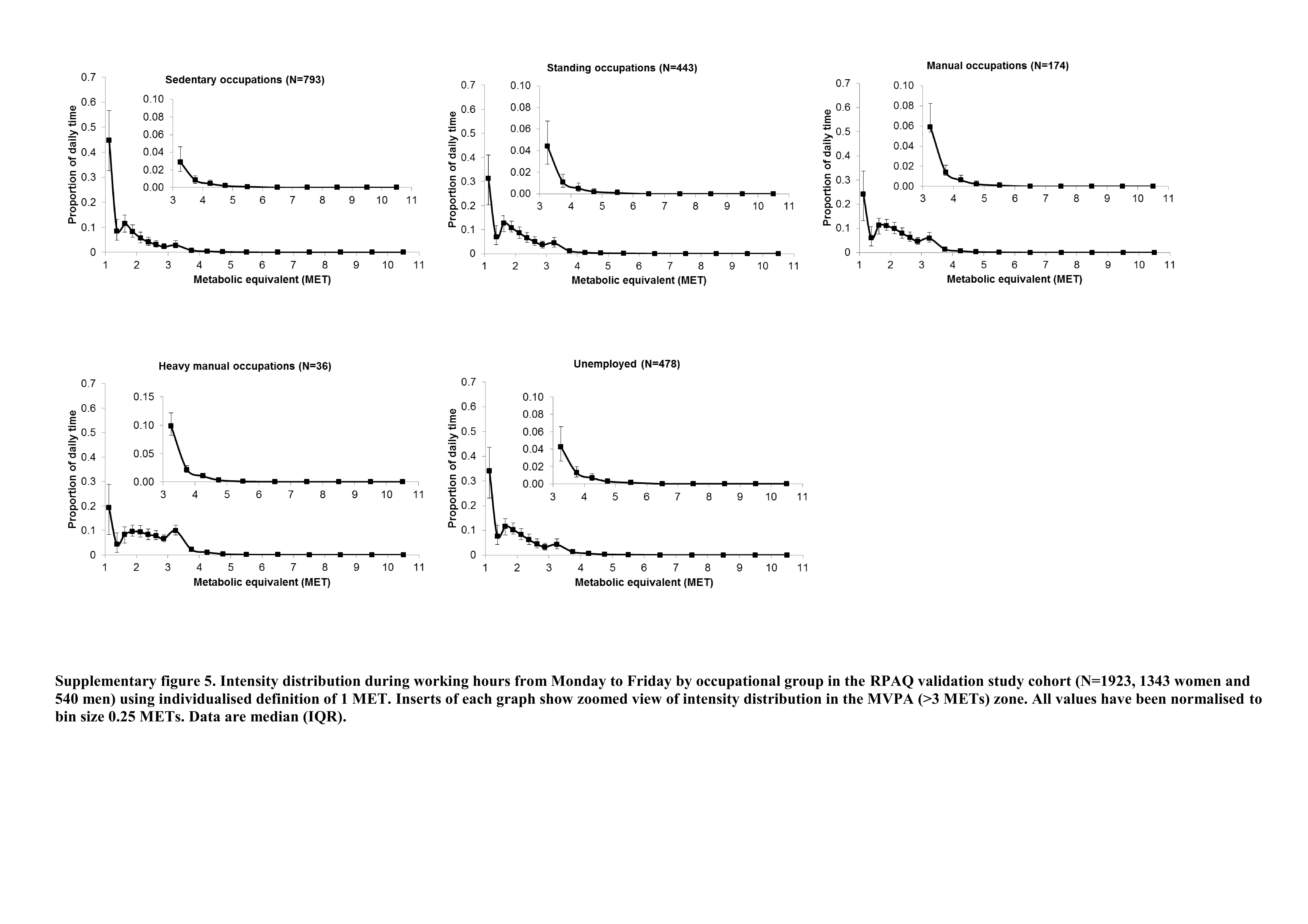

Supplement: Figure S5 — Intensity distribution during working hours from Monday to Friday by occupational group in the RPAQ validation study cohort (N = 1923, 1343 women and 540 men) using individualised definition of 1 MET. Inserts of each graph show zoomed view of intensity distribution in the MVPA (>3 METs) zone. All values have been normalised to bin size 0.25 METs. Data are median (IQR). (TIF) [file pone.0114103.s006.tif]

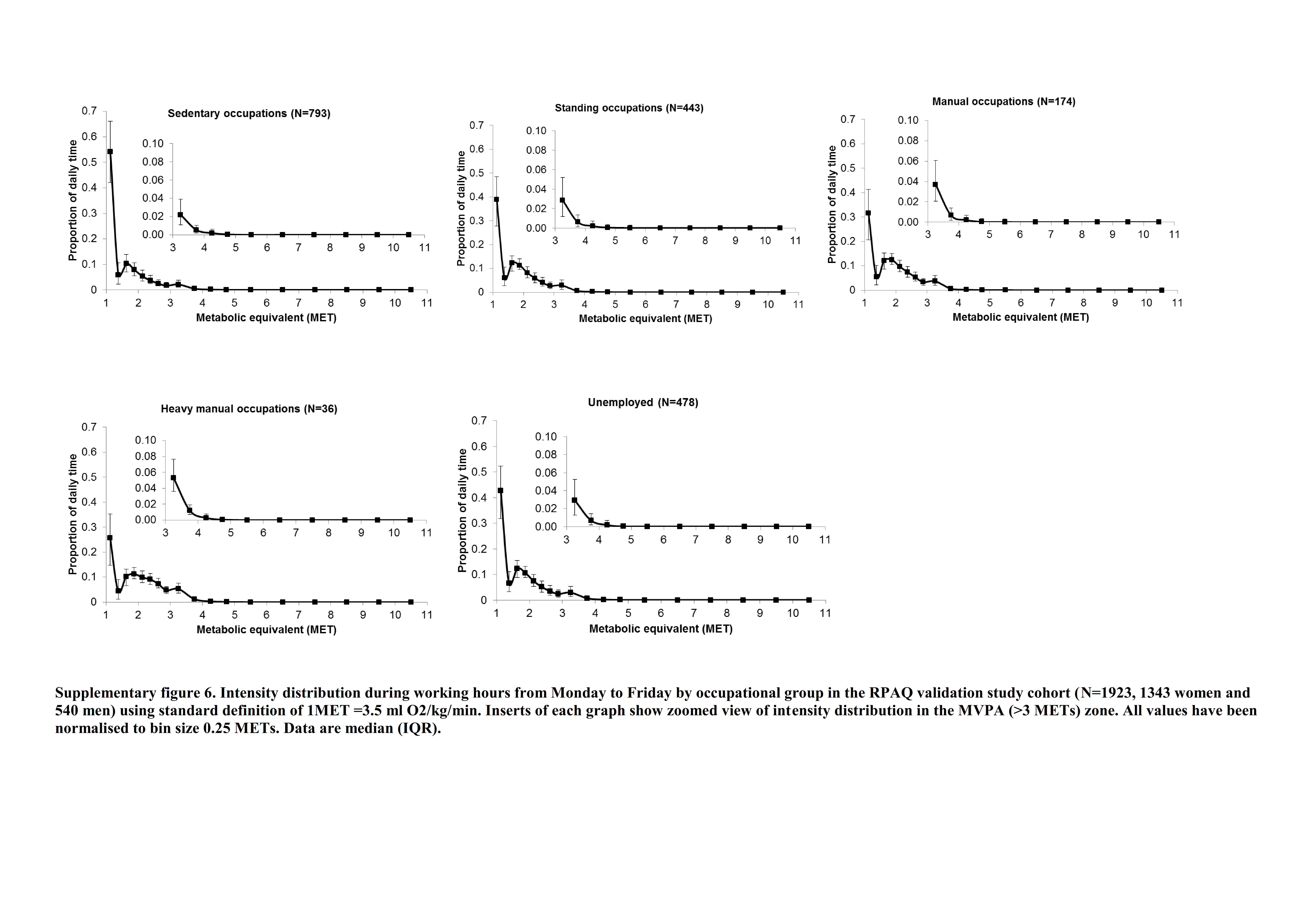

Supplement: Figure S6 — Intensity distribution during working hours from Monday to Friday by occupational group in the RPAQ validation study cohort (N = 1923, 1343 women and 540 men) using standard definition of 1MET = 3.5 ml O2/kg/min. Inserts of each graph show zoomed view of intensity distribution in the MVPA (>3 METs) zone. All values have been normalised to bin size 0.25 METs. Data are median (IQR). (TIF) [file pone.0114103.s007.tif]
